# Rescue of amyotrophic lateral sclerosis phenotype in a mouse model by intravenous AAV9-*ADAR2* delivery to motor neurons

**DOI:** 10.1002/emmm.201302935

**Published:** 2013-09-24

**Authors:** Takenari Yamashita, Hui Lin Chai, Sayaka Teramoto, Shoji Tsuji, Kuniko Shimazaki, Shin-ichi Muramatsu, Shin Kwak

**Affiliations:** 1 CREST, Japan Science and Technology Agency, Graduate School of Medicine, University of TokyoBunkyo-Ku, Tokyo, Japan; 2 Department of Neurology, Graduate School of Medicine, University of TokyoBunkyo-Ku, Tokyo, Japan; 3 Center for Disease Biology and Integrative Medicine, Graduate School of Medicine, The University of TokyoBunkyo-Ku, Tokyo, Japan; 4 Department of Neurosurgery, Jichi Medical UniversityShimotsuke, Tochigi, Japan; 5 Department of Neurology, Jichi Medical UniversityShimotsuke, Tochigi, Japan; 6 Clinical Research Center for Medicine, International University of Health and WelfareIchikawa, Chiba, Japan

**Keywords:** adeno-associated virus (AAV) 9, adenosine deaminase acting on RNA 2 (ADAR2), AMPA receptor, amyotrophic lateral sclerosis (ALS), gene therapy

## Abstract

Amyotrophic lateral sclerosis (ALS) is the most common adult-onset motor neuron disease, and the lack of effective therapy results in inevitable death within a few years of onset. Failure of GluA2 RNA editing resulting from downregulation of the RNA-editing enzyme adenosine deaminase acting on RNA 2 (ADAR2) occurs in the majority of ALS cases and causes the death of motor neurons via a Ca^2+^-permeable AMPA receptor-mediated mechanism. Here, we explored the possibility of gene therapy for ALS by upregulating ADAR2 in mouse motor neurons using an adeno-associated virus serotype 9 (AAV9) vector that provides gene delivery to a wide array of central neurons after peripheral administration. A single intravenous injection of AAV9-ADAR2 in conditional ADAR2 knockout mice (AR2), which comprise a mechanistic mouse model of sporadic ALS, caused expression of exogenous ADAR2 in the central neurons and effectively prevented progressive motor dysfunction. Notably, AAV9-ADAR2 rescued the motor neurons of AR2 mice from death by normalizing TDP-43 expression. This AAV9-mediated ADAR2 gene delivery may therefore enable the development of a gene therapy for ALS.

## INTRODUCTION

Amyotrophic lateral sclerosis (ALS) is the most common adult-onset motor neuron disease. The lack of an effective therapy for ALS results in death from respiratory muscle weakness within a few years of onset. Therefore, the development of effective therapies is eagerly awaited. However, mechanism-based therapeutic strategies have yet to be developed for this disease. More than 90% of ALS cases are sporadic, and the most of them do not carry mutations in genes that are known to be causative in familial forms of ALS. Rather, loss of TDP-43 from the nucleus with abnormal TDP-43-positive cytoplasmic inclusions (TDP-43 pathology) in motor neurons is the pathological hallmark of sporadic ALS (Aizawa et al, 2010). Furthermore, in the majority of the patients with sporadic ALS, considerable proportions of motor neurons in the spinal cord express abnormal glutamine/arginine (Q/R) site-unedited GluA2 (a subunit of the α-amino-3-hydroxy-5-methylisoxazole-4-propionic acid (AMPA) receptor (Kawahara et al, 2004; Kwak & Kawahara, 2005)) because of reduced expression of an RNA editing enzyme called adenosine deaminase acting on RNA 2 (ADAR2) (Aizawa et al, 2010; Hideyama et al, 2012). Analysis of conditional ADAR2 knockout mice (ADAR2^flox/flox^/VChAT-Cre.Fast or AR2 mice) demonstrated that insufficient ADAR2 expression induced the death of motor neurons via an abnormal Ca^2+^-permeable AMPA receptor-mediated mechanism (Hideyama et al, 2010). Notably, the expression of abnormal Ca^2+^-permeable AMPA receptors causes TDP-43 pathology in motor neurons through activation of calpain, a Ca^2+^-dependent serine protease, and the resultant expression of aggregation-prone TDP-43 fragments (Yamashita et al, 2012a). The concomitant occurrence of reduced ADAR2 levels and TDP-43 pathology in the same motor neurons (Aizawa et al, 2010) and the presence of calpain-dependent abnormal TDP-43 fragments in the brains and spinal cords of sporadic ALS patients (Yamashita et al, 2012a) suggest that the molecular cascade observed in AR2 mice is similar to what occurs in the motor neurons of ALS patients and that normalization of ADAR2 activity represents a potential therapeutic strategy for ALS patients (Aizawa et al, 2010; Yamashita et al, 2012a, 2012b).

We attempted to broadly deliver ADAR2 cDNA to motor neurons to enhance ADAR2 activity to a level sufficient to edit the Q/R site of GluA2 pre-mRNA. Recently, clinical challenges for gene therapy have been observed when using adeno-associated virus (AAV) as a vector (Lonergan et al, 2005; Mingozzi & High, 2011). Because motor neurons are widely localized in the cranial motor nerve nuclei in the brainstem and the anterior horns of the entire spinal cord, systemic injection of a vector is preferable to local injection to provide global gene delivery to the motor neurons of ALS patients. Although the blood–brain barrier presents a hurdle to the delivery of genes to the central nervous system from the periphery, several groups have been successful in transducing AAV serotype 9 (AAV9) to foetal, neonatal and adult animal motor neurons in the spinal cord via intravenous administration (Benkhelifa-Ziyyat et al, 2013; Dayton et al, 2012; Duque et al, 2009; Foust et al, 2009). To achieve widespread and selective expression of the *ADAR2* gene in motor neurons and avoid off-target delivery, we used AAV9 as a vector and the synapsin I (SYNI) promoter for neuron-specific expression of ADAR2 cDNA (Supporting Information Fig S1; Iwata et al, 2013).

## RESULTS

### Examination of ADAR2 activity in cells and mouse brains using AAV9-hADAR2

**Figure 1:**
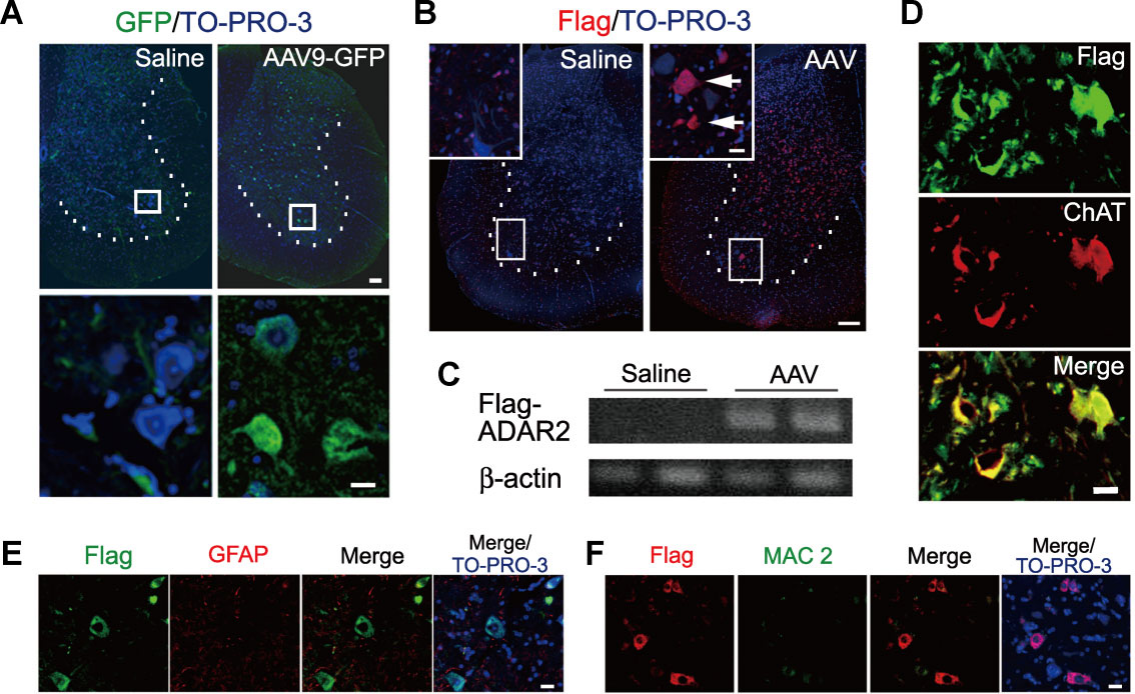
Gene delivery to mouse cortical and spinal neurons using AAV9 vectors Large motor neurons (anterior horn cells, AHCs) expressed GFP in the spinal cords of wild-type mice injected in the tail vein with AAV9-GFP (1.5 × 10^11^ vg/body, *n* = 4). Scale bars, 50 µm (upper panels) and 20 μm (lower panels).Expression of Flag protein was observed in the AHCs of AR2 mice injected with AAV9-Flag-hADAR2 in the tail vein (AAV; 2.1 × 10^12^ vg/body) but not in mice injected with saline (Saline). Arrows indicate Flag-positive AHCs. Scale bars, 50 and 20 μm (insets).RT-PCR demonstrated the expression of Flag-hADAR2 in the anterior horn of AAV9-treated AR2 mice.Representative immunofluorescence images of the spinal cords of AR2 mice treated with AAV9-Flag-hADAR2 showing the expression of Flag (green) and choline acetyltransferase (ChAT) (red) protein using anti-Flag and anti-ChAT antibodies. Magnified view of the boxed areas in Supporting Information Fig S3A. Scale bar, 20 μm.Flag-hADAR2 did not colocalize with GFAP. TO-PRO-3 was used as a cell marker. Scale bar, 20 μm.Flag-hADAR2 did not colocalize with Mac2. TO-PRO-3 was used as a cell marker. Scale bar, 20 μm.

We constructed cDNA encoding N-terminally Flag-tagged wild-type (WT) human ADAR2a (hADAR2) with the SYNI promoter. After confirming the expression of active hADAR2 protein in Neuro2a cells transduced with AAV9-Flag-hADAR2 (Supporting Information Fig S2; Nishimoto et al, 2008), we confirmed the expression of AAV9-delivered hADAR2 at an effective level *in vivo* by injecting AAV9-hADAR2^E396A^, an inactive hADAR2 mutant, directly into the cerebral cortex of WT mice. Because the extensive RNA editing at known ADAR2-specific positions in normal mouse brains (Nishimoto et al, 2008) masked the effects of exogenous ADAR2 expression, we used ADAR2^E396A^ and evaluated the efficacy of gene delivery based on reductions in editing efficiency. The extent of RNA editing at the two ADAR2-specific positions, *i.e*. the Q/R site of the GluA2 pre-mRNA and the K/E site of the cytoplasmic fragile X mental retardation protein interacting protein 2 (CYFIP2) mRNA, was significantly decreased at the site of injection of AAV9-hADAR2^E396A^ but not in the contralateral hemisphere, remote regions (ipsilateral hemisphere 2.0–2.5 mm posterior to the injection site), or brain regions injected with AAV9-GFP (Supporting Information Fig S2D). Delivery of genes packaged in AAV9 into the large anterior horn cells (AHCs) in the mouse spinal cord was confirmed by injection of AAV9-SYNI-GFP (1.5 × 10^11^ vg/body) into the tail vein of 6-week-old WT mice (*n* = 4/group). Approximately 20% of AHCs were immunoreactive for GFP (Fig 1A).

### Gene delivery to cortical and spinal neurons using AAV9 vectors

Consistently, expression of Flag protein and mRNA was demonstrated in the brains and spinal cords (Fig 1B–D and Supporting Information Figs S1 and S3), including the AHCs (Fig 1B and Supporting Information Fig S3B), of AR2 mice injected with AAV9-Flag-hADAR2 in the tail vein (Fig 1B and C). In addition, proliferation of both activated astrocytes showing increased GFAP immunoreactivity and MAC2-positive activated microglial cells was not detected in the spinal cords, including the regions around AAV9-infected neurons of WT mice injected with AAV9-GFP and of AR2 mice injected with AAV9-Flag-hADAR2 (Fig 1E, F and Supporting Information Figs S4 and S5). There was no significant expression of Flag protein in the peripheral organs, as previously reported (Iwata et al, 2013). These results indicate that the intravenously injected AAV9 vector delivers the *ADAR2* gene to neurons and expression of the delivered *ADAR2* in neurons is not toxic without inducing abnormal glial cell reaction.

### Behavioural changes in AR2 mice intravenously injected with AAV9-Flag-hADAR2

Next, to test whether a therapeutic level of ADAR2 expression could be achieved through systemic administration, AAV9-SYNI-Flag-hADAR2 was intravenously injected into AR2 mice (2.1 × 10^12^ vg/body) in the pre-symptomatic stage (*n* = 16; 9–13 weeks old) or after they began exhibiting motor dysfunction to model therapy for patients (*n* = 5; 15 weeks old). Age-adjusted AR2 mice injected with saline were used as controls. AR2 mice provide a mechanistic mouse model of sporadic ALS (Hideyama et al, 2010; Yamashita et al, 2012a). These mice undergo a progressive decline in motor function resulting from the progressive loss of ADAR2-lacking AHCs over a period ranging from 2–3 months to 6–8 months of age (Hideyama et al, 2010); thus, if effective expression of the *ADAR2* gene is achieved by AAV9-hADAR2 infection, motor dysfunction should be ameliorated through the prevention of the death of the AHCs. Mice were observed for behaviour every week until the end point, which was set as the time point at which they failed to stay on the rotarod for more than 10 s or their age exceeded 36 weeks.

**Figure 2:**
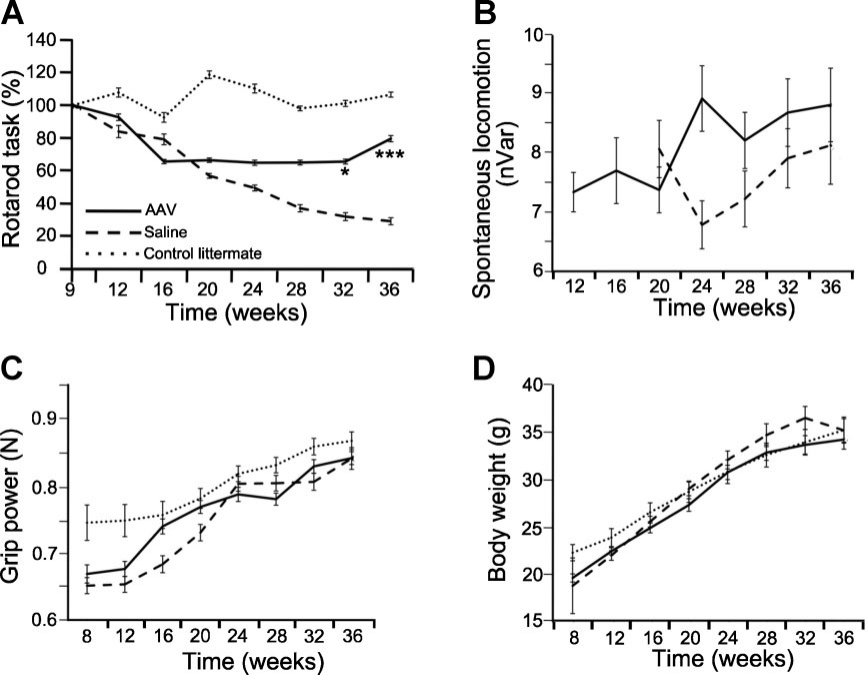
Behavioural changes in AR2 mice intravenously injected with AAV9-Flag-hADAR2 **A.** Rotarod performance was significantly better in AR2 mice injected with the AAV9 vector (AAV; *n* = 16) than in saline-treated AR2 mice (Saline; *n* = 13). *p < 0.05, ***p < 0.0001 (Student's *t*-test against Saline). All error bars represent the s.e.m.**B.** Spontaneous locomotion was not significantly different between the AAV (*n* = 9) and Saline (*n* = 4) groups. All error bars represent the s.e.m.**C,D.** Grip power (C) and body weight (D) did not significantly differ between the AAV (*n* = 16) and Saline (*n* = 13) groups. All error bars represent the s.e.m. Control littermate: Cre-negative, normal littermate mice (*n* = 5). Each plot represents the average value of 4 weeks, and the numbers on the abscissa are the postnatal week indicated as the last week of the 4-week period. Each plot in (A) and (B) represents the value of the latency to fall relative to the value at 9 weeks of age. All error bars represent the s.e.m.

Treatment with AAV9-Flag-hADAR2 ameliorated or virtually completely prevented the progressive decline in rotarod performance observed in saline-treated AR2 mice (Fig 2A). This effect on rotarod performance was also evident in AR2 mice treated after the initiation of decline (Supporting Information Fig S6). AAV9-injected AR2 mice exhibited higher spontaneous locomotor activity than saline-injected AR2 mice, but the difference between the two groups did not reach statistical significance (Fig 2B). The grip power and body weight did not differ between the AAV9-injected and saline-injected AR2 mice (Fig 2C and D). The failure to rescue grip power may be related to the milder motor dysfunction of forelimbs compared to hindlimbs.

### Rescue of motor neurons from death

**Figure 3:**
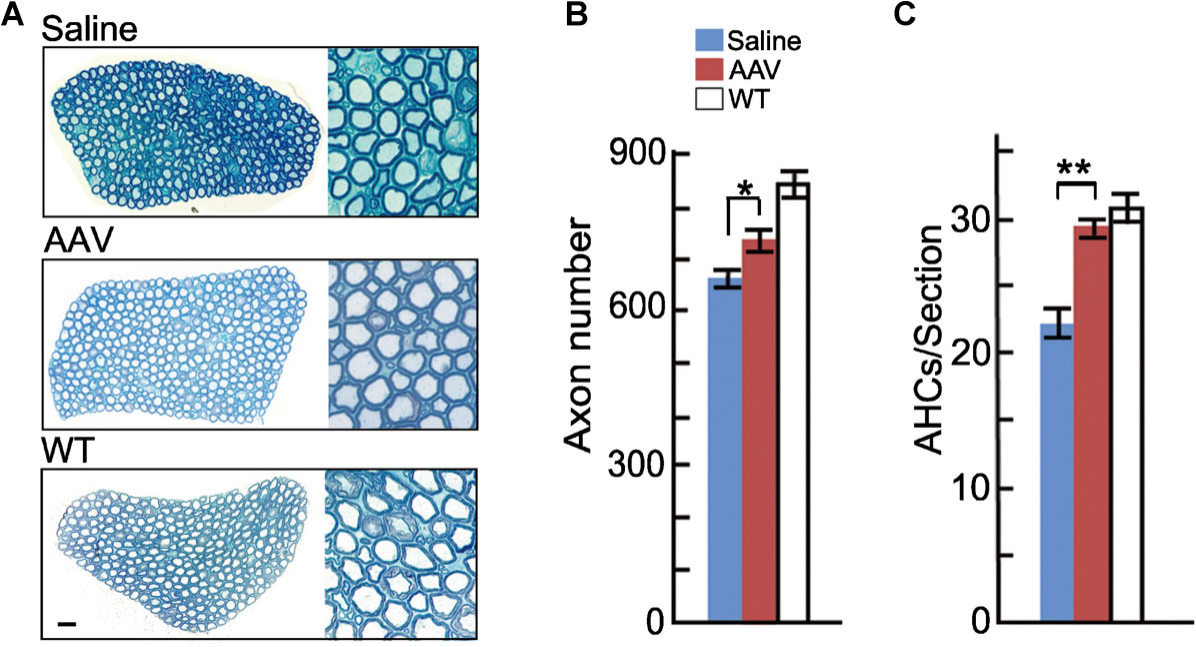
Rescue of motor neurons from death The ventral root of the fifth lumbar segment (L5). Saline: a saline-injected AR2 mouse, AAV: a pre-symptomatically AAV9-Flag-hADAR2 injected AR2 mouse (AAV), WT: a wild-type mouse (39 weeks of age). Inset; magnified view. Scale bar: 20 and 64 µm for the insets.Numbers of axons in the ventral root in Saline (649.8 ± 17.8, *n* = 10; blue column), AAV (733.0 ± 28.6, *n* = 5; red column), and WT (840.0 ± 26.5, *n* = 3; white column) groups. All error bars represent the s.e.m. *p < 0.05 (Student's *t*-test).Number of AHCs in L5 (*n* = 5 for each group). All error bars represent the s.e.m. **p < 0.01 (Student's *t*-test). Numbers of AHCs in the unilateral AH are presented. Symbol colours are the same as in B.

To confirm that systemic injection of AAV9-hADAR2 prevented the degeneration of AHCs in the AR2 mice, we counted the axons in the ventral root of the fifth lumbar spinal segment (L5) and the number of AHCs in the L5. The number of remaining axons and AHCs was significantly higher in AAV9-injected AR2 mice than in control AR2 mice (Fig 3 and Supporting Information Fig S7). These results indicate that degeneration of AHCs and the resulting motor dysfunction in AR2 mice were effectively prevented by AAV9-mediated delivery of ADAR2.

### Rescue of motor neuron functions

**Figure 4:**
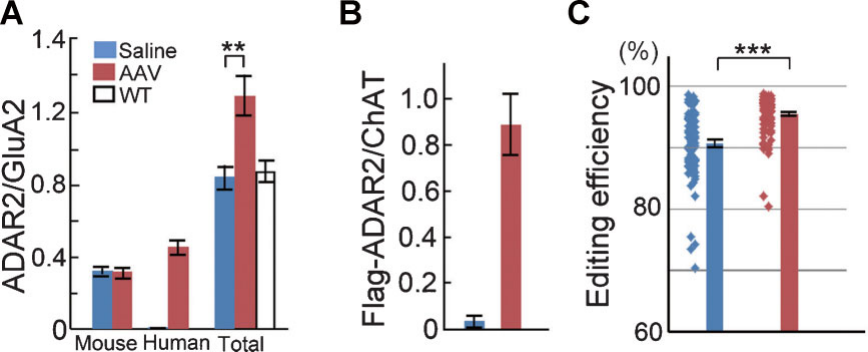
ADAR2 gene expression in the anterior horn of the spinal cord (AH) The relative abundance of mouse ADAR2 mRNA did not significantly differ between AR2 mice injected with AAV9-Flag-hADAR2 (AAV) and those injected with saline (Saline; *n* = 5 for each group). hADAR2 mRNA was expressed at a significant level in AAV. The relative abundance of total (human and mouse) ADAR2 mRNA was 1.5-fold higher in AAV (*n* = 4) than in Saline (*n* = 4). **p < 0.01 (Mann–Whitney *U*-test). All error bars represent the s.e.m.The relative abundance of hADAR2 mRNA standardized with mouse ChAT mRNA indicated that there was significant expression of hADAR2 in ChAT-positive AHCs in AAV. *n* = 15 (AAV) and *n* = 6 (Saline). All error bars represent the s.e.m.The editing efficiency at the GluA2 Q/R site in the AH lysates (four samples from each mouse) from the AAV group (*n* = 8) was significantly higher than that in the control group (*n* = 7) (95.20 ± 0.36% vs. 90.68% ± 0.55%; Mann–Whitney *U*-test, ***p < 0.0001; (*p* = 0.0128 when compared between AAV and saline groups)). All error bars represent the s.e.m.

Because rescue of death of AHCs likely results from restoration of ADAR2 activity in the motor neurons of AR2 mice (Hideyama et al, 2010), we next investigated whether the expression and activity of ADAR2 were increased in the motor neurons after systemic injection of AAV9-hADAR2. The relative abundance of mouse ADAR2 did not significantly differ between the AAV9-injected and saline-injected AR2 mouse spinal cords (Fig 4A) but AAV9-Flag-hADAR2 infection induced 1.5-fold increase in the expression level of total ADAR2 mRNA in the spinal cords (Fig 4A) and brains (Supporting Information Fig S8). Messenger RNA of both hADAR2 and choline acetyltransferase (ChAT) was demonstrated in the spinal cord lysates of AAV9-injected AR2 mice (Fig 4B) and the editing efficiency at the GluA2 Q/R site was significantly higher in the remaining motor neurons of AAV9-injected AR2 mice than in those of the control AR2 mice (Fig 4C). These results indicated that hADAR2 was delivered to and functioned in motor neurons. However, ADAR2 protein level did not significantly differ among AAV9-injected AR2 mice, saline-injected AR2 mice and WT mice (Supporting Information Fig S8B and C). The failure to detect the difference despite of the difference in the ADAR2 activity was presumably due to the fact that a modest increase in the ADAR2 expression level with preservation of death of 10–20% of motor neurons in AAV-treated AR2 mice may be masked in ADAR2 expressed in the remaining motor and non-motor neurons and other cells in the anterior horn.

**Figure 5:**
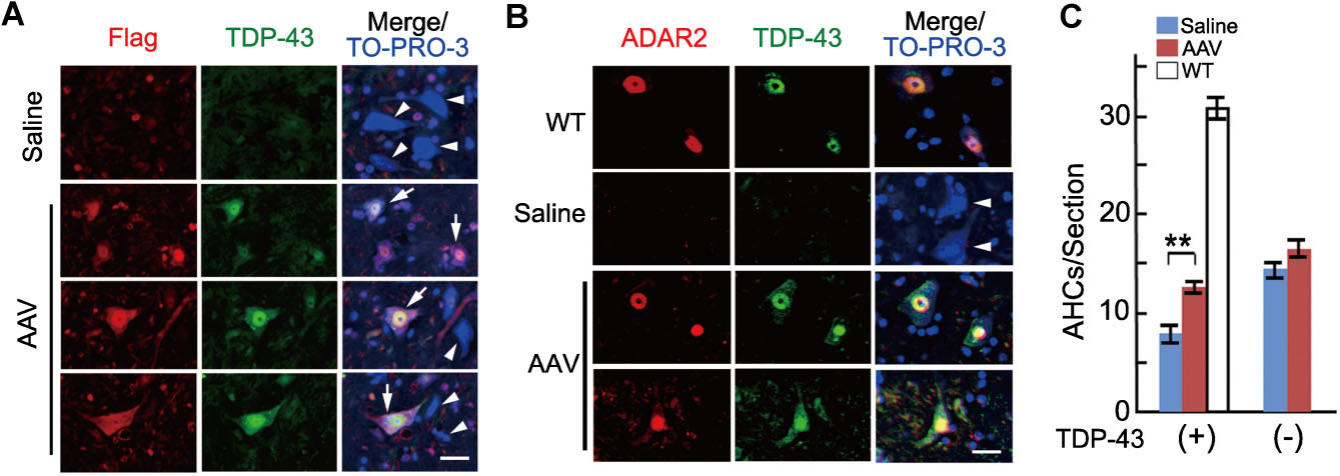
Rescue of TDP-43 in motor neurons Immunohistochemistry for TDP-43 (green) and Flag (red) in the spinal cord. AHCs were recognized as large (≥20 µm diameter) and TO-PRO-3-positive (blue) cells in the AH. Flag-positive AHCs exhibited intense nuclear and faint cytoplasmic TDP-43 immunoreactivity (arrows) in AAV9-injected AR2 mice (AAV). Arrowheads indicate AHCs negative for both Flag immunoreactivity and TDP-43 immunoreactivity in the nucleus in saline-injected AR2 mice (Saline) and AAV. TO-PRO-3 was used as a cell marker. Scale bar is 20 μm.Double immunostaining of the AH for TDP-43 and ADAR2. The nuclei of the AHCs were either double-positive or double-negative for TDP-43 (green) and ADAR2 (red) in AAV, Saline and wild-type mice (WT). Scale bar is 20 μm.Number of TDP-43-positive and -negative AHCs in Saline (blue columns), AAV (red columns) and WT (white column) (*n* = 5 for each group). All error bars represent the s.e.m. **p < 0.01 (Student's *t*-test).

Consistent with the effective prevention of the death of AHCs, loss or mislocalization of TDP-43 in the AHCs of saline-treated AR2 mice was rescued in AAV9-injected AR2 mice, and Flag-expressing AHCs exhibited predominantly nuclear TDP-43 localization (Fig 5 and Supporting Information Fig S9). Indeed, the number of AHCs showing normal nuclear localization of TDP-43 was markedly increased in the AAV9-injected AR2 mice compared with the control AR2 mice (Fig 5C and Supporting Information Fig S10). These results indicate that AAV9-mediated delivery of hADAR2 is sufficient to upregulate ADAR2 activity at the GluA2 Q/R site, associated with normalization of TDP-43 pathology in ADAR2-lacking AHCs, thereby preventing neuronal death.

## DISCUSSION

In this study, we explored the possibility of using gene therapy to treat ALS by enhancing ADAR2 activity through delivery of the ADAR2 gene to mouse motor neurons using AAV9 as a vector, together with the SYNI promoter to achieve neuron-specific expression of the ADAR2 gene. We showed that AAV9-ADAR2 was successfully delivered to and functioned in motor neurons (Figs 1D and 4 and Supporting Information Fig S3), with a virtual absence of peripheral expression being detected following i.v. administration in the mice. Furthermore, the expression of the delivered ADAR2 prevented the progression of motor dysfunction and neuronal death with restoring ADAR2-mediated RNA editing (Figs 2–4 and Supporting Information Fig S7) without inducing any adverse effects in neurons or surrounding tissues (Fig 1 and Supporting Information Figs S4 and S5) in AR2 mice, which provide a mechanistic model of sporadic ALS (Hideyama et al, 2010; Yamashita et al, 2012a). Notably, effective prevention of the progressive decline in rotarod performance was observed, even after the manifestation of motor dysfunction (Supporting Information Fig S6). Because the death of ADAR2-deficient AHCs is prevented by the expression of Q/R site-edited GluA2 in the absence of ADAR2 in homozygous and heterozygous AR2 mice (Hideyama & Kwak, 2011; Hideyama et al, 2010, 2012), our results indicate that AAV9-mediated delivery of hADAR2 enables the restoration of ADAR2 activity in ADAR2-deficient AHCs to a level that is sufficient to edit the Q/R sites of virtually all of the GluA2 pre-mRNAs expressed in AR2 mice (Fig 4 and Supporting Information Fig S8). Given that the level of ADAR2 reduction is more modest in the motor neurons of patients with sporadic ALS (Hideyama et al, 2012; Kawahara et al, 2004) compared to the AHCs of AR2 mice in which ADAR2 is absent (Hideyama et al, 2010), a therapeutic level of ADAR2 activity would be more easily achieved in ALS patients using this method of gene delivery.

In the AAV9-treated AR2 mice, TDP-43 immunoreactivity was observed to be predominantly nuclear in Flag-positive AHCs, as in normal mouse AHCs, which is in marked contrast to the absence of TDP-43 immunoreactivity or the presence of numerous cytoplasmic TDP-43 immunoreactive aggregates found in ADAR2-lacking AHCs from untreated AR2 mice (Fig 5 and Supporting Information Fig S9). TDP-43 pathology in motor neurons of the spinal cord is the pathological hallmark of ALS (Aizawa et al, 2010; Arai et al, 2006; Neumann et al, 2006), and mislocalization of TDP-43 is induced by exaggerated calpain-mediated cleavage of TDP-43 into aggregation-prone fragments, although further activation of calpain cleaves the aggregation-prone fragments into smaller soluble fragments (Yamashita et al, 2012a). The abnormal activation of calpain is a consequence of an increased Ca^2+^ influx through Ca^2+^-permeable AMPA receptors containing Q/R site-unedited GluA2 in the ADAR2-deficient AHCs of AR2 mice, and expression of edited GluA2, even in the absence of ADAR2, rescues TDP-43 mislocalization and AHC loss by normalizing calpain activity (Yamashita et al, 2012a). Therefore, it is likely that ADAR2 delivery normalized the subcellular localization of TDP-43 in the AHCs by reducing Ca^2+^ influx through AMPA receptors and the resulting calpain activation. Our results demonstrating the AAV9-ADAR2-mediated normalization of TDP-43 provide additional evidence that ADAR2 deficiency represents a cause of ALS.

Delivery and expression of target genes has been achieved using AAV9 with the cytomegalovirus (CMV) or CMV enhancer/beta-actin (CB) promoter in many cell types including neurons (Benkhelifa-Ziyyat et al, 2013; Thevenot et al, 2012), but off-target overexpression of genes may have deleterious effects. Here, using our neuron-specific AAV9-SYNI vector, we were successful in limiting target gene expression to neurons with a virtual absence of expression in peripheral tissues. Recent reports in human patients have shown that the effects of gene delivery by direct injection of AAV2 vectors into the brain continue for up to 12 months without serious adverse effects (Hwu et al, 2012), and we found strong and long-term expression of *ADAR2* in mouse motor neurons. The ADAR2 transgenic mice were phenotypically normal except for moderate obesity (Singh et al, 2007), which suggests that expression of the exogenous ADAR2 gene is relatively safe.

Taken together, our results indicate that therapy through replacement of deficient ADAR2 protein is a logical approach to therapeutic intervention for ALS that could be effective in the majority of ALS patients and that given the demonstrated effects and safety of the clinical use of AAV, gene therapy using AAV9-hADAR2 is a promising therapeutic strategy for ALS. The appropriate route of administration and the required dose of AAV should be determined in future studies. The results from our ALS model mice and recently reported results from Alzheimer's disease model mice (Iwata et al, 2013) suggest that AAV vector-mediated cDNA delivery to central neurons through the vasculature using neuron-specific promoters as regulators of gene expression is a new therapeutic approach for the treatment of neurodegenerative diseases.

## MATERIALS AND METHODS

### Antibodies

The primary antibodies were as follows: rabbit anti-human beta-actin (IMGENEX Corp.); goat anti-ADAR2 (E-20) (Santa Cruz Biotech.); rabbit anti-GFP (Cell Signaling Technology, Danvers); rabbit anti-DYKDDDDK tag (Flag) (Cell Signaling Technology); goat anti-DDDDK tag (Abcam); sheep anti-rat RED1 (ADAR2, Exalpha Biologicals, Inc.); rabbit anti-glial fibrillary acidic protein (GFAP) (Lab Vision); rat anti-mouse MAC-2 (Cedarlane); goat anti-choline acetyltransferase (ChAT) (Millipore); and rabbit anti-human TDP-43 (10782-1-AP, ProteinTech Group, Inc., designated as TDP-43).

The secondary antibodies used for immunohistochemistry were as follows: Alexa Fluor 488 goat anti-rat IgG; Alexa Fluor 488 chicken anti-rabbit IgG; Alexa Fluor 555 donkey anti-goat IgG; Alexa Fluor 555 goat anti-rabbit IgG; Alexa Fluor 555 goat anti-rat IgG; Alexa Fluor 555 donkey anti-sheep IgG (Invitrogen); and the HRP-DAB System (Vector Co.). Peroxidase-conjugated goat anti-rabbit IgG and peroxidase-conjugated horse anti-mouse IgG (Cell Signaling Technology, Inc., Danvers) were used for immunoblot analyses.

### Reagents

TO-PRO-3 was purchased from Invitrogen.

### Construction of plasmids

To generate full-length human ADAR2 expression constructs, we first amplified the coding region from human ADAR2a cDNA (hADAR2) generated from the human HeLa cell line using the primers ADAR2UP1 (5′-AAAAAGAATTCATGGATATAGAAGATGAAGAAAAC-3′) and ADAR2DW1 (5′-AAAAATCTAGATCAGGGCGTGAGTGAGAACTGGTCC-3′). After gel purification, the PCR products were digested with *Eco*RI and *Xba*I and cloned into a pCI mammalian expression vector (Promega, Madison, WI) that had been digested with the same restriction enzymes. The construct of Flag-hADAR2 was amplified using PCR with primers specific for Flag-ADAR2UP1 (5′-AAAAAGCTAGCTCCACCATGGATTACAAGGATGACGACGATAAGATCGATATAGAAGATGAAG-3′) and Flag-ADAR2DW1 (5′-AAAAAGGTACCTCAGGGCGTGAGTGAGAAC-3′). The resultant products were digested with *Nhe*I and *Kpn*I and cloned into pCI (Promega). Site-directed mutagenesis of hADAR2 was conducted to substitute Ala for Glu (E396A) using a KOD Plus mutagenesis kit (Toyobo, Japan). All constructs were verified by DNA sequencing.

### Cell culture and transfection

Neuro2a cells were cultured in DMEM high-glucose medium (WAKO, Tokyo, Japan) supplemented with 10% foetal bovine serum (Invitrogen, Carlsbad, CA, USA), 100 U/ml penicillin and 100 µg/ml streptomycin (Invitrogen) in 5% CO_2_ at 37°C. The culture medium was changed once after 24 h and then every 2 days. The cells were grown in 6-well plates at a density of 3.5 × 10^4^ cell/cm^2^. The cultured cells were transfected with 2.5 µg of expression plasmid using Lipofectamine LTX and PLUS™ reagents (Invitrogen). The cells were cultured for 72 h and then harvested.

### Production of recombinant AAV vectors

The AAV vector plasmids contained an expression cassette consisting of the mouse synapsin I (SYNI) promoter, followed by the cDNA of interest, a woodchuck hepatitis virus posttranscriptional regulatory element, and a simian virus 40 polyadenylation signal sequence between the inverted terminal repeats of the AAV3 genome. The AAV9 vp cDNA was synthesized, and the sequence was identical to that previously described (Gao et al, 2004) except for the substitution of thymidine for adenine 1337, which introduced an amino acid change from tyrosine to phenylalanine at position 446 (Petrs-Silva et al, 2011). Recombinant AAV vectors were produced by transient transfection of HEK293 cells using the vector plasmid, an AAV3 rep and AAV9 vp expression plasmid, and the adenoviral helper plasmid pHelper (Agilent Technologies, Santa Clara, CA) as described previously (Li et al, 2006). The recombinant viruses were purified by isolation from two sequential continuous CsCl gradients, and the viral titres were determined by qRT-PCR. The viral vectors used for expression of hADAR2 (AAV9-hADAR2), an inactive mutant hADAR2 with an E396A amino acid substitution (AAV9-hADAR2^E396A^), Flag-tagged hADAR2 (AAV9-Flag-hADAR2 and AAV9-Flag-hADAR2^E396A^) and green fluorescent protein (AAV9-GFP), contained the entire cDNA sequences of *ADARB1* (GenBank accession number NM_015833 and NM_015834) or *EGfp*.

### Animals

The animals used in this study were homozygous conditional ADAR2 knockout mice (*ADAR2*^*flox/flox*^/VAChT-Cre.Fast; AR2). In AR2 mice, *Adarb1* encoding ADAR2 is conditionally targeted in motor neurons using the Cre/loxP system, and ADAR2 activity is completely ablated in approximately 50% of motor neurons, which are therefore unable to edit the Q/R site of GluA2. AR2 mice display slowly progressing motor dysfunction resulting from a loss of spinal AHCs. Because reduced GluA2 Q/R site-editing occurs in motor neurons in sporadic ALS, AR2 mice recapitulate the molecular pathology of sporadic ALS.

Recombinant AAV vectors were injected intravenously into mice via the mouse tail vein. In some experiments (Supporting Information Fig S4), AAV vectors were injected in the left ventricle of the heart of mice anaesthetized with pentobarbital (50 mg/kg, i.p.) over a period of 1 min with a 0.5 ml syringe equipped with a 29-gauge needle. Homozygous AR2 mice (*n* = 16) were injected with AAV9-hADAR2 vectors (2.14 × 10^12^ vg/body) before (*n* = 11) or after (*n* = 5) the initiation of motor dysfunction that was defined by a decline in rotarod performance. Age-adjusted AR2 mice were used as disease controls, and WT or Cre-negative ADAR2^flox^ mice of the same strain were used as normal controls. All studies were performed in accordance with the Guidelines for Animal Studies of the University of Tokyo and NIH. The Committee on Animal Handling at the University of Tokyo also approved the experimental procedures. AR2 mice injected with AAV9-hADAR2 vectors before the initiation of motor dysfunction were used for immunohistochemistry and biochemical analyses at 36 weeks of age (*n* = 5–8). In some experiments, WT mice of the same strain at 10 weeks of age was injected with AAV9-GFP (7.2 × 10^11^ vg/body) and histologically observed 11 weeks after the injection.

### Behavioural analyses

We determined the maximal time before falling in a mouse-specific rotarod (Muromachi Kikai Co. LTD MK-610A). After training at 10 rpm, mice were placed on the rotarod turning at a speed of 4 rpm, and the speed of the rotarod was then accelerated to 40 rpm linearly over 120 s. Each run consisted of three trials, and the maximum value of the three runs was recorded. We defined disease onset as the time point at which the mice exhibited <75% of the average performance of the initial 3 weeks for three consecutive trials. The end point was set as the point at which the falling time was <10 s for 3 consecutive weeks or the mice reached 36 weeks of age. Grip power was measured using a dynamometer (NS-TRM-M; Neuroscience Corp.). Spontaneous activity data from individual mice were collected using a piezoelectric sensor sheet (Biotex) as previously reported by Nakamura et al (2008). Mice were housed in cages on a 12 h:12 h light-dark cycle with free access to food and water. Piezoelectric sensor sheets placed under the cages for 24 h were used to measure the daily activity of the mice. The sensor sheets' output voltage signals were proportional to the pressure generated by the activity of the mice. The signals were sampled at 100 Hz with 16-bit resolution after passing through a 0.5–50 Hz band-pass filter and then digitized and stored on a computer every minute. To evaluate spontaneous activity, we calculated the average of 24 h of data and recorded this value. All behavioural measurements were conducted weekly by a researcher, who was blind to the virus administration condition, genotype and age of the mice.

### Western blot analysis

Neuro2a cells and frozen tissues were homogenized by sonication in 20 volumes of extraction buffer (50 mM HEPES, pH 7.5, 1 mM EDTA, 100 mM NaCl, 10 mM DTT and 0.1% CHAPS). The homogenate was centrifuged at 1000 g for 10 min at 4°C. The supernatant was boiled with 4 × SDS gel loading buffer and subjected to SDS–PAGE. After electrophoresis, proteins were transferred to an Immobilon-P transfer membrane (Millipore, Bedford, MA), and immunoblotting for ADAR2, Flag and beta-actin was conducted. Goat anti-ADAR2 (E-20) (Santa Cruz Biotech.) (1:2000), rabbit anti-DYKDDDDK Tag (Flag) (Cell Signaling Technology) (1:2000), and rabbit anti-beta-actin (IMGENEX Corp.) (1:2000) were used as primary antibodies, and peroxidase-conjugated goat anti-rabbit IgG (Cell Signaling Technology, Inc.) (1:2000) and peroxidase-conjugated affinipure donkey anti-goat IgG (H + L) (Jackson ImmunoResearch, West Grove, PA) (1:2000) were used as secondary antibodies. Visualization was conducted using ECL plus Western blotting detection reagents (GE Healthcare Bioscience, Piscataway, NJ, USA). Specific bands were detected using a LAS 3000 system (Fujifilm, Tokyo).

**The paper explained**

**PROBLEM:**

ALS is the most common adult-onset motor neuron disease in which motor neurons innervating skeletal muscles selectively and progressively undergo degeneration from undetermined mechanism. The progressive nature of the disease leads the patients with ALS to death from failure of respiratory muscles within a few years of onset without effective therapy. Recently, with the progress of pathogenic mechanism of ALS, several potential target molecules for therapy have been demonstrated. However, because motor neurons are localized widely in the nuclei of cranial motor nerves and the spinal cord, global delivery of therapeutic agents to the motor neurons is required to accomplish therapeutic effects. Although delivery of the therapeutic agents through the vasculature enables widespread delivery, the blood–brain-barrier prevents entrance of molecules from blood to the brain and spinal cord. Therefore, safe delivery of therapeutic agents widely to motor neurons using appropriate vehicles is required for development of ALS therapy.

**RESULTS:**

Progressive death of the motor neurons in the brains and spinal cords cause ALS phenotype and expression of abnormal GluA2 (a subunit of the AMPA receptor that is involved in the neuronal excitation in the brain and spinal cord) with glutamine residue at the glutamine/arginine (Q/R) site (GluA2Q) is a disease-specific and potentially death-causing molecular abnormality occurring in the motor neurons of the patients with sporadic form of ALS that accounts for the majority of ALS patients. Motor neurons normally express Ca^2+^-impermeable AMPA receptors containing GluA2 with arginine residue at the Q/R site (GluA2R) in the assembly, but express Ca^2+^-permeable AMPA receptors when GluA2Q is expressed, which leads motor neurons to death. Given that this event would be closely relevant to ALS etiology, we attempt to develop a therapeutic strategy for ALS by broadly delivering cDNA of ADAR2, the enzyme that converges GluA2Q to GluA2R, to motor neurons in the aim to enhance the expression of normal GluA2R. To achieve widespread and selective expression of the *ADAR2* gene in motor neurons through a peripheral route avoiding off-target delivery, we used a viral vector AAV9 and the neuron-specific SYNI promoter. A single intravenous injection of AAV9-ADAR2 in AR2 mice, which comprise a mechanistic mouse model of sporadic ALS, effectively prevented progressive motor dysfunction and death of motor neurons by enhancing ADAR2 activity. Notably, AAV9-ADAR2 normalized the abnormal expression profile of TDP-43, which is the ALS-specific pathological change, in the remaining motor neurons. Thus, our therapeutic strategy semi-permanently normalized disease phenotype, neuronal death and the disease-specific molecular marker in the ALS model mice.

**IMPACT:**This is the first report on successful pre-clinical ALS therapy based on plausible pathogenic mechanism, achieving semi-permanent therapeutic effects on a mechanistic disease mouse model. Potency of the therapy on the model mice and safety as demonstrated by the clinical use in some other diseases provide delivery of the *ADAR2* gene using AAV9 with SYNI promoter as potential therapy applicable to patients with ALS. Intravenous route of delivery using a relatively safe vehicle would facilitate clinical trials.

### RNA extraction and reverse transcription

Total RNA was isolated from the cells and spinal cords of mice using an RNeasy micro kit (Qiagen) and trizol (Invitrogen) and treated with DNaseI as recommended by the manufacturer. First-strand cDNA was synthesized from the total RNA using a Onestep RT-PCR kit (Qiagen), Ready-To-Go You-Prime First-Strand Beads (GE Healthcare Bioscience) and 50 ng of random primers (Invitrogen) as recommended by the manufacturer.

### Analysis of the conversion of adenosine to inosine at the GluA2 Q/R site and CYFIP2 K/E site editing

The efficiency of the conversion of adenosine to inosine in the GluA2 mRNA, pre-mRNA and the CYFIP2 mRNA was calculated using a Bioanalyzer 2100 (Agilent Technologies) following the digestion of PCR products with restriction enzymes (Bhalla et al, 2004; Kawahara et al, 2003, 2004). The amplified GluA2 mRNA and pre-mRNA PCR products were digested with *Bbv*I (New England Biolabs, Ipswich, MA). The amplified CYFIP2 mRNA PCR products were digested with *Mse*I (New England Biolabs).

The PCR products from edited GluA2 pre-mRNA molecules contain one intrinsic *Bbv*I recognition site, whereas the products originating from the unedited GluA2 contain an additional recognition site. Therefore, digestion of the PCR products from the edited GluA2 pre-mRNA and GluA2 mRNA should produce two bands (129 and 71 bp, pre-mRNA; 200 and 44 bp, mRNA), whereas digestion of bands originating from the unedited GluA2 pre-mRNA or mRNA molecules should produce three bands (91, 38 and 71 bp, pre-mRNA; 119, 44 and 81 bp, mRNA). The density of the 71- or 44-bp band, which results from digestion of both the edited and unedited pre-mRNA or mRNA, and the 129- or 200-bp band, which is solely the product of the edited pre-mRNA or mRNA, were quantified and the editing efficiency was calculated as the ratio of former to the latter for each sample (Nishimoto et al, 2008; Sawada et al, 2009). Similarly, *Mse*I digestion of the RT-PCR product generated from edited CYFIP2 yields two bands (117 and 209 bp), whereas that generated from unedited CYFIP2 mRNA yields three bands (117, 60 and 149 bp) (Nishimoto et al, 2008). The PCR primers used in these assays are provided in Supporting Information Table S1.

### Real-time quantitative polymerase chain reaction

Quantitative PCR was performed using a LightCycler System (Roche Diagnostics, Indianapolis, IN). Standards and cDNA samples were amplified in a reaction mixture (20 µl total volume) composed of 10 µl of 2× LightCycler 480 Probes Master Roche (Roche Diagnostics), each primer at 0.5 µM and the Universal Probe Library (Roche Diagnostics) at 0.1 µM. We determined the expression level of ADAR2 mRNA using different primer pairs for mouse ADAR2, human ADAR2 and total (both human and mouse) ADAR2 cDNA (Supporting Information Table S2). The reaction was initially incubated at 95°C for 10 min, and amplification of the templates was performed with a denaturing step at 95°C for 10 s and a primer annealing step at 60°C for 30 s. As an internal control, the expression of human β-actin was also measured in each sample using a LightCycler Primer/Probe Set (Roche Diagnostics; Supporting Information Table S2) and the same PCR conditions (Sawada et al, 2009; Yamashita et al, 2012c).

### Immunohistochemistry

Under deep anesthesia with isoflurane mice, were transcardially perfused with 3.5% paraformaldehyde and 0.5% glutaraldehyde in phosphate-buffered saline (PBS). The brains and spinal cords were removed and immersed in serially increasing concentrations of sucrose–PBS solutions (final sucrose concentration of 30%). The sucrose-immersed spinal cords were cut at a thickness of 10 µm with a cryostat (Model LEICA CM1850; Leica). The sections were immunostained with a standard avidin–biotin–immunoperoxidase complex method using VECTASTAIN ABC IgGs (Vector Co.) for the secondary antibodies. Rabbit anti-TDP-43 (ProteinTech Group, Inc., 1:100) and sheep anti-rat RED1 (ADAR2; Exalpha Biologicals, Inc., 1:100) were used for the primary antibodies. Colour was developed using the HRP-DAB System (Vector Co.).

Immunofluorescent staining of the sections was performed using rabbit anti-TDP-43 (ProteinTech Group, Inc., 1:200) and sheep anti-rat RED1 (ADAR2; Exalpha Biologicals, Inc., 1:200) as the primary antibodies. Sections were then incubated with Alexa Fluor 555 donkey anti-sheep IgG (Invitrogen, 1:200) and Alexa Fluor 488 chicken anti-rabbit IgG (Invitrogen, 1:200), respectively, as the secondary antibodies. The sections were examined under an LSM-510 confocal microscope (Zeiss) after nuclear staining with 0.5 µM TO-PRO-3 for 30 min.

### Morphological observation

Mice were killed by overdose of isoflurane, and the brains and spinal cords were removed and then either quickly frozen on dry ice (right hemisphere and the first cervical to the second lumbar spinal cord segments) or fixed with 3.5% paraformaldehyde and 1% glutaraldehyde in PBS (left hemisphere and the rest of the spinal cord). Frozen samples were stored at −80°C until use. Paraformaldehyde-fixed samples were immersed in the same fixative overnight and then rinsed in PBS. Sections of the fixed fifth lumbar (L5) spinal cord segment were sequentially immunostained for Flag (Flag-hADAR2) and TDP-43 using the immunofluorescence system. The fluorescent images were analysed using a fluorescence microscope (BIOREVO BZ-9000; Keyence Corp, Osaka, Japan). Large AHCs with diameters larger than 20 µm were separately counted for each mouse. TDP-43-positive AHCs (in the ventral grey matter ventral to the line running though the ventral edge of the central canal) were counted in four L5 sections for each mouse. The ventral roots of L5 were then postfixed in 1% phosphate-buffered osmium tetroxide. The signal intensity of TDP-43 was examined using Image J software. TDP-43-positive AHCs were counted when the signal intensity was more than threefold higher than the background. After three washes with phosphate buffer, each sample was dehydrated in a graded series of ethanol and embedded in Epon (Wako). Thin sections (1 µm) of the L5 ventral root were stained with 0.1% toluidine blue, digitized using a BIOREVO BZ-9000 (Keyence), and axons were counted manually by a researcher who was blind to the virus injection condition.

### Tyramide signal amplification (TSA)

The perfusion-fixed, sucrose-immersed spinal cords were cut to a thickness of 12 µm using a cryostat (Model LEICA CM1850; Leica). The sections were incubated with 3.0% H_2_O_2_ in PBS and TNB blocking buffer [0.1 M Tris–HCl, pH 7.5, 0.15M NaCl and 0.5% blocking Reagent (PerkinElmer)]. Sections were serially incubated with goat anti-choline acetyltransferase (ChAT) (Millipore; 1:2000 in TNB blocjing buffer) at 4°C overnight, incubated with HRP-conjugated donkey anti-goat IgG (Abcam, 1:2000) for another 1 h at room temperature and finally incubated with tetramethylrhodamine plus amplification tyramide reagent (1:250 in amplification solution) for 20 min at room temperature. After washing, the sections were subjected to the next round of immunohistochemistry after blocking with 3.0% H_2_O_2_ in PBS. The sections were incubated with the rabbit anti-Flag antibody (Cell Signaling Tech; 1:200) in Can Get Signal buffer A (Toyobo) at 4°C overnight and then serially with HRP-conjugated chicken anti-rabbit IgG (Abcam, 1:2000) in Can Get Signal buffer A (Toyobo) for another 1 h at room temperature and with fluorescein plus amplification tyramide reagent (1:250 in amplification solution) for 20 min at room temperature. The sections were examined under a BIOREVO BZ-9000 microscope (Keyence Corp, Osaka, Japan) after nuclear staining with 0.5 µM TO-PRO-3 for 60 min. Bars represent 50 or 20 µm.

### Statistical analysis

Average data are presented as means and s.e.m. Statistical analyses were conducted using JMP 9 software (SAS Institute, Inc.). For statistical comparisons of two groups, we used unpaired, two-tailed Student's *t* tests or Mann–Whitney *U* tests. Differences were considered significant when *p* < 0.05.

## Supplementary material

As a service to our authors and readers, this journal provides supporting information supplied by the authors. Such materials are peer reviewed and may be re-organized for online delivery, but are not copy-edited or typeset. Technical support issues arising from supporting information (other than missing files) should be addressed to the authors.

**Figure S1. Viral transduction of the AAV9-SynI vector in the mouse.** Western blot analyses using an anti-Flag-tag antibody showed a widespread distribution of the AAV9-SynI vector in the central nervous system, including the spinal cord, cortex, cerebellum and olfactory bulb of AR2 mice intravenously injected with AAV9-Flag-hADAR2 (AAV). Saline; AR2 mice treated with saline.

**Figure S2. Expression of AAV-delivered ADAR2 in Neuro2a cells and wild-type mouse brains.** RT-PCR (A) and Western blot analyses (B) of ADAR2 demonstrated that hADAR2 was expressed in Neuro2a cells infected with AAV9-hADAR2 or AAV9-hADAR2E396A. (C) The editing efficiency at the CYFIP2 mRNA K/E site was significantly higher in Neuro2a cells infected with AAV9-hADAR2 compared with those infected with AAV9-GFP- or AAV9-hADAR2E396A (mean ± s.e.m.; *n* = 6 for each group). ***p* < 0.01 (Mann–Whitney *U*-test). D. Editing efficiencies at the Q/R site of GluA2 pre-mRNA and the K/E site of CYFIP2 mRNA in the brains of wild-type mice injected with AAV9-hADAR2E396A in the cerebral cortex. Editing efficiencies were significantly decreased at the injection site, but not in the remote ipsilateral (2.0–2.5 mm posterior to the injection site) or contralateral hemisphere. **p* < 0.05, ****p* < 0.0001 (Mann–Whitney *U*-test,). The editing efficiencies at these sites were not changed in the mouse brain area injected with AAV9-GFP. All error bars represent the s.e.m., *n* = 4–12.

**Figure S3. Expression of AAV9-Flag-hADAR2 in the AR2 mouse spinal cord after intravenous injection.** Immunohistochemistry for Flag (green) and ChAT (red) in the spinal cords of an AR2 mouse intravenously injected with AAV9-Flag-hADAR2. The boxed areas represent the area for high power magnification image of Fig 1D. Scale bar, 50 mm. (B and C) Magnified view of the dotted line areas in (A). Both ChAT-positive anterior horn cells (AHCs) (B) and ChAT-negative dorsal horn cells (DHCs) (C) exhibit Flag immunoreactivity. Scale bars, 20 mm. (D) Numbers of AHCs classified by immunoreactivity to Flag and ADAR2 in AR2 mice intravenously injected with AAR9-Flag-hADAR2 or saline. AR2 mice (*n* = 4 for each group) were examined at 33–36 weeks of age, ****p* < 0.001 (Student's *t*-test). All error bars represent the s.e.m.

**Figure S4. GFAP and MAC2 immunoreactivity in the spinal cords of wild-type mice injected with AAV9-GFP.** Axial section of the spinal cord of wild type mice (21 weeks of age) 11 weeks after the intravenous injection with AAV9-GFP (7.2 × 10^11^ vg/body). There was little or virtually no increase of GFAP and MAC2 immunoreactivity in the spinal cord. There was no GFAP-positive astrocytes or MAC2-positive microglia around the GFP-positive neurons (bottom). Scale bars, 50 mm (upper panels) and 20 mm (bottom panels).

**Figure S5. GFAP and MAC2 immunoreactivity in the spinal cord of AAV-injected AR2 mice.** In contrast to the intense GFAP and MAC2 immunoreactivity in the spinal anterior horns (AH) of the saline-treated AR2 mice (Saline), there was virtually no increase of the immunoreactivity in the AH of AR2 mice intravenously injected with AAV9-Flag-hADAR2 (AAV). Scale bars, 50 mm (upper panels) and 20 mm (bottom panels).

**Figure S6. Rotarod performance of AAV-injected AR2 mice.** Rotarod performance was significantly better in AR2 mice intravenously injected with AAV9-Flag-hADAR2 at presymptomatic stages (*n* = 11; AAV) than in saline-treated AR2 mice (*n* = 13; Saline) (Student's *t*-test against Saline group, **p* < 0.05, ****p* < 0.0001). All error bars represent the s.e.m. (B) Injection of the AAV vector into AR2 mice that already exhibited declined rotarod performance prevented the progression of motor dysfunction (*n* = 5; AAV; Student's *t*-test against Saline group, ***p* < 0.01, ****p* < 0.0001). All error bars represent the s.e.m.

**Figure S7. Magnified view of the ventral roots of the fifth lumber segment (L5).** Each panel is the ventral root of either saline-injected (Saline, *n* = 3) or AAV9-Flag-hADAR2-injected AR2 mice (AAV, *n* = 3). Arrowheads indicate axonal degeneration. Scale bar: 10 mm.

**Figure S8. Effects on the expression of ADAR2 in the brain and spinal cord.** The relative abundance of hADAR2 mRNA was significantly higher in the brains of AR2 mice injected with AAV9-Flag-hADAR2 (AAV) (*n* = 3) than in those of saline-injected AR2 mice (Saline) (*n* = 3), **p* < 0.05 (Mann–Whitney *U*-test). All error bars represent the s.e.m. (B and C) Immunoblot analysis of brain (B) and spinal cord extracts (C) demonstrated that the expression of ADAR2 protein did not significantly differ between AAV and Saline groups.

**Figure S9. Rescue of TDP-43 in motor neurons.** Immunohistochemistry for TDP-43 (green) and Flag (red) in the right half of the spinal cord of AR2 mice intravenously injected with AAV9-Flag-hADAR2. The boxed areas represent the area for high power magnification image of Fig 5A. Scale bar, 50 mm. (B) There were AHCs devoid of TDP-43 immunoreactivity (arrowheads) and those exhibiting numerous TDP-43-positive cytoplasmic aggregates without nuclear TDP-43 immunoreactivity (double arrowheads) in the saline-treated AR2 mice (Saline). Flag-positive AHCs exhibited intense nuclear and faint cytoplasmic TDP-43 immunoreactivity (arrows) in AAV9-Flag-ADAR2-injected AR2 mice (AAV). Scale bar is 20 mm.

**Figure S10. The signal intensity of TDP-43-positive and -negative AHCs.** Immunohistochemistry for TDP-43 (green) and Flag (red) in the spinal cord. AHCs were recognized as large (≥20 μm diameter) and TO-PRO-3-positive (blue) cells in the AH. The panels represent the enlarged image of Fig 5A. The profile images showed fluorescence intensity of TDP-43 along the yellow line. In Fig 5C, we designated AHCs as TDP-43-positive when the signal intensity was more than threefold higher (>60) than the background (average ± s.e.m., 8.80 ± 0.12; max 20).

**Table S1.** Primer sequences.

**Table S2.** TaqMan probes used for quantitative polymerase chain reaction.
