# Supplementary material for: Rescue of amyotrophic lateral sclerosis phenotype in a mouse model by intravenous AAV9-ADAR2 delivery to motor neurons
Source: EMBO Mol Med. 2013 Sep 24;5(11):1710–9. doi: 10.1002/emmm.201302935 (PMC3840487; doi:10.1002/emmm.201302935)
Supplement: Supplementary file 1 [file emmm0005-1710-SD1.pdf]

Rescue of amyotrophic lateral sclerosis phenotype in a mouse model by intravenous AAV9-ADAR2 delivery to motor neurons

Takenari Yamashita, Hui Lin Chai, Sayaka Teramoto, Shoji Tsuji, Kuniko Shimazaki, Shin-ichi Muramatsu and Shin Kwak

Corresponding author: Shin Kwak, Graduate School of Medicine, University of Tokyo

Review timeline:

Submission date:	22 April 2013
Editorial Decision:	25 April 2013
Appeal received:	25 April 2013
Editorial Decision:	06 June 2013
Revision received:	18 July 2013
Additional Editorial Correspondence:	17 August 2013
Additional Author Correspondence :	20 August 2013
Editorial Decision:	20 August 2013
Revision received:	23 August 2013
Accepted:	23 August 2013

Transaction Report:

(Note: With the exception of the correction of typographical or spelling errors that could be a source of ambiguity, letters and reports are not edited. The original formatting of letters and referee reports may not be reflected in this compilation.)

Editor: Céline Carret

1st Editorial Decision 25 April 2013

Thank you for the submission of your manuscript "Rescue of amyotrophic lateral sclerosis phenotype in a mouse model by intravenous AAV9-ADAR2 delivery to motor neurons."

I have now had the opportunity to carefully read your paper and the related literature and I have also discussed it with my colleagues. I am afraid that we concluded that the manuscript is not well suited for publication in EMBO Molecular Medicine and have therefore decided not to proceed with peer review.

We appreciate that your study reports on a successful gene therapy for ALS in a mouse model. Restoration of ADAR2 expression in neurons of pre-symptomatic or post-symptomatic conADAR2-KO mice results in improved motor functions (but without restoring grip power) in animals and prevented motor neuron death by normalizing TDP-43 expression. However, the underlying molecular pathway on which this therapy is based was previously reported. More importantly, gene therapy improving ALS phenotype was shown before using different genes in different mouse models. Therefore, while the study will certainly be of interest to the immediate community, given the existing literature, the conceptual advance provided by the study is not sufficient for further consideration in EMBO Molecular Medicine.

I am sorry that I could not bring better news this time.

Appeal received 25 April 2013

Thank you for reviewing thouroughly our manuscript.

I would like to draw your attention in the following points:

1) Our conditional ADAR2 knockout mice is only the mechanistic model for the sporadic ALS that accounts for the majority of ALS patients.
Previous ALS model mice used for gene therapy are for SOD1-associated familial ALS and SOD-associated ALS differ from sporadic ALS in the absence of TDP-43 pathology and lack of ADAR2 down-regulation. The majority of ALS researchers now consider that the two diseases have different etiology despite that the phenotype is similar (therefore the disease name ALS is used for these two different diseases). Therefore, the therapeutic strategy for these two diseases must be different and transgene of the SOD1 gene does not rescue sporadic ALS even if is rescues SOD1-associated ALS. Please understand that it is not right to say "gene therapy improving ALS phenotype was shown before using different genes in different mouse models" .

2) Our study aims to provide evidence that our method of the ADAR2 gene delivery works in the patients and scoping clinical trial on patients with ALS. Because there is no effective therapy for ALS and cure of patients with ALS, particularly sporadic ALS, is what people eagerly desire.
We demonstrated therapeutic effects our strategy using unique mechanistic mouse mode for sporadic ALS, which may provide patients with hope.

I appreciate it very much if you could reconsider the possibility of publishing our results in EMBO Mol Med.

2nd Editorial Decision 06 June 2013

Thank you for the submission of your manuscript to EMBO Molecular Medicine. We have now heard back from the three referees whom we asked to evaluate your manuscript. Although the referees find the study to be of potential interest, they also raise a number of concerns that need to be fully addressed in the next final version of your article.

As you will see from the enclosed reports, referee 1 makes very detailed and clear suggestions to improve the conclusiveness of the data. While referees 2 and 3 are more concise, they also ask for additional controls and fuller presentation of the results as the findings are currently minimally presented and the discussion is insufficient, obscuring the primary data.

In our view the suggested revisions would render the manuscript much more compelling and interesting to a broad readership. We therefore hope that you will be prepared to undertake the recommended experimental revision.

Please note that that it is our journal's policy to allow only a single round of revision, and that acceptance or rejection of the manuscript will therefore depend on the completeness of your response and the satisfaction of the referees with it.

I look forward to seeing a revised form of your manuscript as soon as possible.

***** Reviewer's comments *****

Referee #1 (General Remarks):

Yamashita et al. reported a therapeutic study to test the effect of Adar2 gene delivery to conditional ADAR2 KO (AR2) mice which showed ALS-like phenotype. The authors found that a single intravenous injection of AAV9-ADAR2 improved motor function in rotarod task, motor neuronal loss, axonal degeneration, GluA2 editing activity, and TDP-43 pathology. It is noteworthy that a single injection could reverse ALS-phenotype in AR2 mice in regard to the therapeutic possibility; however the manuscript is overall immature and remains many issues to be addressed.

Major Critiques
1.The author's group reported that AR2 mice also showed abnormal Ca permeability, activation of calpain, and TDP-43 fragmentation (Hideyama et al., 2010, Yamashita et al., 2012). It is recommended to demonstrate the rescue effects on them by refilling ADAR2 expression.
2.It is unclear what the ratio of Flag-ADAR2-positive AHCs was and how much mRNA/protein of ADAR2 were recovered after the AAV treatment. The authors previously reported that approximately half of AHCs in the spinal cord were knocked out for ADAR2 gene in AR2 mice (Hideyama et al., 2010). This study lacks the information of AHCs with ADAR2 staining. It is highly recommended to show the ratio of neurons recovered with ADAR2 expressions by injection. The readers may also wonder what happened in AHCs without KO as well as non-motor neurons after Flag-ADAR2 overexpression. Did they show twice or more amount of ADAR2 expression than WT did? If so, was excess of ADAR2 expression beneficial or harmful for AHCs? To address these questions, the precise quantification of ADAR2 expression for AHCs and other neurons in CNS is necessary.
The authors actually presented mRNA levels of Adar2 mRNA in Fig 4A by qPCR instead of immunohistochemistry. Although the right bar of total ADAR2 seems that AAV injection achieved twice amounts of ADAR2 mRNA as saline control, the way of measurement is not appropriate. The amounts of mRNA levels measured by different primers, different sets of primers for mouse and human Adar2, are not addable when calculated by the ratio to the reference gene. It is necessary to use the set of primers which are able to recognize both mouse and human ADAR2. As in the sophisticated data of their previous work (Fig2 in Hideyama et al., 2010), the single-cell-based analysis using microlaserdisection would be the most desirable way to show the precise levels of ADAR2 together with the activity of GluR2 Q/R site-editing.

3.Control must be injection of AAV9-mock or AAV9-GFP instead of saline only. The authors stated in Fig 1E-F that proliferation of both activated astrocytes showing increased GFAP immunoreactivity and MAC2-positive activated microglial cells was detected in the anterior horns of AR2 mice injected with AAV9- Flag-hADAR2. This could be due to AAV virus injection. From this point of view, control should be AAV virus injection.

4.Two injection groups were applied for the study: 11 animals for the injection at the pre-symptomatic stage and 5 for the injection after the onset. It is confusing since Fig 2A, C, D, and E were for the entire group but only Fig 2B showed the group of the injection after the onset. Fig 2 must be shown separately for each group.

5.The age of mice used in Fig3-5 must be indicated. It is also necessary to specify which treatment group of mice was used, injection before or after the onset.

6.Although the age of mice in Fig3 is not specified, the numbers of AHCs/section in Fig3C seem to be apparently discrepant from their previous work (Fig4C in Hideyama et al., 2010). The previous work found the numbers of AHCs per section less than 20 at the age of 2 months. It seems that the age of mice using in Fig3 was near or at the end point that was 9 months.

7.The criteria for TDP-43 positive AHCs in Fig 5C are uncertain. The signal quantification is necessary.

8.In Discussion section (P8, L7-10), the authors stated that "Furthermore, the expression of the delivered ADAR2 prevented the progression of motor dysfunction and neuronal death by

normalizing the Ca2+ influx through AMPA receptors and downstream molecular events leading to death, such as calpain activation with resultant mislocalization of TDP-43 in AR2 mice (Fig 3, 4 and 5)". There was no such data of calcium influx or calpain in the manuscript.

Minor Critiques

1.In P5 L15 what are "remote regions"?
2.Wild-type should be added in Fig 3A, 3C, 4A, and 5C.
3.The description in P6 L16, "apparently, although not significantly, improved" is not scientific.
4.In P12 L12, "2.14x10^-12" must be "2.14x10^-12 VG/body".
5.TO-PRO-3 staining was used for detecting the entire AHCs in Fig5. TO-PRO-3 is basically a nuclear staining method. The reason must be mentioned or it would be better to be replaced with a standard neuronal-staining marker.
6.There are several sentences with no space before parenthesis.

Referee #2 (Comments on Novelty/Model System):

Please see review.

Referee #2 (General Remarks):

The authors have determined the ability of ADAR2 expressed from an AAV vector to restore editing function in neurons in a model for ALS, in which endogenous ADAR2 knockout out in motor neurons. If fully validated, this is an important observation.

The critical data appear in Figure 2 in which the authors show that AAV-ADAR2 expression restores rotarod performance in the mouse model. A motor task, and prevents further decline when administered late.

In Figure 1D, Flag-ADAR2 appears to be expressed in neurons that are not CHAT positive. How widespread is the expression?

Figure 4 present the data on the effect of ADAR2 expression in the mouse model on GlyA2 mRNA. It would help to show examples of the original PCR data. The bar graphs are hard to interpret. Specifically, what is the meaning of the statement (Figure 4 legend) that "the relative abundance of hADAR2 mRNA was significantly higher in AR2 mice injected with AAV9-Flag-hADAR2 than in saline injected controls"? There should be absolutely no human ADAR2 mRNA in mouse and if there is a signal, it is possible that the PCR assay is in error. In Figure 4C, there is only a modest change in editing efficiency with AAV expression of ADAR2. How can such a small change account for the big recovery of rotarod function seen in Figure 2?

Referee #3 (Comments on Novelty/Model System):

The authors consider that the downregulation of adenosine deaminase acting on RNA1 (ADAR2) is a general pathogenic mechanism in Amyotrophic Lateral Sclerosis. TDP43 accumulation in the cytoplasm is, in their view, related to the expression of abnormal Ca2+-permeable AMPA receptor through activation of calpain. The hypothesis is interesting but has been developed and tested only by the research group of the authors and awaits confirmation from other teams.

Referee #3 (General Remarks):

The authors hypothesize that the downregulation of adenosine deaminase acting on RNA1 (ADAR2) is a general pathogenic mechanism in Amyotrophic Lateral Sclerosis (ALS). TDP43 accumulation in the cytoplasm is, in their view, related to the expression of abnormal Ca2+-

permeable AMPA receptor through activation of calpain. To test further their hypothesis, they treated ADAR2 conditional KO mice wich develop a motor deficit (AR2 mice)with an AAV9 vector containing human ADAR2 gene. The treatment prevented othe neuronal loss and the loss of axons. The paper is clear. I would however recommend providing larger and higher quality histological pictures. Fig 1, E and F are barely visible.
In fig 2, a bar should indicate that the significant difference was between the saline treated and the AAV treated animal. In fig 3 A, no histological difference is visible between saline and AAV treated animals. The density of myelin fibers appears to remain very high in the AR2 mice. A better view of the histological sections should demonstrate the myelin loss. In fig 4,C I believe that the n used to compute the SEM is the number of samples (4 samples by mouse for 8 and 7 individuals per group). This explains the very low SEM and the statistical significance between the groups despite the small difference between the means. I believe the correct methodology would be to compute mean per individual, to calculate a SEM using these (mean) individual values with n=8 and 7 respectively. I am not convinced that the difference would remain significant. Fig 5,A TDP43 IHC is negative in the nuclei but there is apparently no labelling in the cytoplasm in the saline treated animals. Was this expected? Is there no inclusion in the cytoplasm as in ALS? Could the authors provide a better view of the pathological and rescued anterior horn cells (larger picture, higher magnification) ?

1st Revision - authors' response 18 July 2013

In line with the comments from editors and reviewers, we have provided additional data to present the results more clearly with sufficient discussion in the revised manuscript. Below, we provide responses (beginning at each arrow) that specifically address each of the reviewers' comments, which are italicized.

To Reviewer #1:

Referee #1 (General Remarks):

Yamashita et al. reported a therapeutic study to test the effect of Adar2 gene delivery to conditional ADAR2 KO (AR2) mice, which showed ALS-like phenotype. The authors found that a single intravenous injection of AAV9-ADAR2 improved motor function in rotarod task, motor neuronal loss, axonal degeneration, GluA2 editing activity, and TDP-43 pathology. It is noteworthy that a single injection could reverse ALS-phenotype in AR2 mice in regard to the therapeutic possibility; however the manuscript is overall immature and remains many issues to be addressed.

Major Critiques
1.The author's group reported that AR2 mice also showed abnormal Ca permeability, activation of calpain, and TDP-43 fragmentation (Hideyama et al., 2010, Yamashita et al., 2012). It is recommended to demonstrate the rescue effects on them by refilling ADAR2 expression.

→ (Response) We thank the reviewer for deep understanding of our work. We have demonstrated that the level of Ca^{2+} influx through AMPA receptors determines the calpain activity and cleavage of TDP-43 (Yamashita et al, 2012, Nat Commun). In response to this comment, we added figures showing TDP-43 mislocalization was rescued in the AHCs expressing Flag-hADAR2 in the AR2 mice treated with AAV9-Flag-hADAR2 in Supporting Information Fig. S9. The description in the results section (page 7 line 7 from the bottom) has been amended accordingly as follows.

"Consistent with the effective prevention of the death of AHCs, loss or mislocalization of TDP-43 in the AHCs of saline-treated AR2 mice was rescued in AAV9-injected AR2 mice, and Flag-expressing AHCs exhibited predominantly nuclear TDP-43 localization (Fig 5 and Supporting Information Fig S9). Indeed, the number of AHCs showing normal nuclear localization of TDP-43 was markedly increased in the AAV9-injected AR2 mice compared with the control AR2 mice (Fig 5C). "

2.It is unclear what the ratio of Flag-ADAR2-positive AHCs was and how much mRNA/protein of ADAR2 were recovered after the AAV treatment. The authors previously reported that

approximately half of AHCs in the spinal cord were knocked out for ADAR2 gene in AR2 mice (Hideyama et al., 2010). This study lacks the information of AHCs with ADAR2 staining. It is highly recommended to show the ratio of neurons recovered with ADAR2 expressions by injection. The readers may also wonder what happened in AHCs without KO as well as non-motor neurons after Flag-ADAR2 overexpression. Did they show twice or more amount of ADAR2 expression than WT did? If so, was excess of ADAR2 expression beneficial or harmful for AHCs? To address these questions, the precise quantification of ADAR2 expression for AHCs and other neurons in CNS is necessary.
The authors actually presented mRNA levels of Adar2 mRNA in Fig 4A by qPCR instead of immunohistochemistry. Although the right bar of total ADAR2 seems that AAV injection achieved twice amounts of ADAR2 mRNA as saline control, the way of measurement is not appropriate. The amounts of mRNA levels measured by different primers, different sets of primers for mouse and human Adar2, are not addable when calculated by the ratio to the reference gene. It is necessary to use the set of primers which are able to recognize both mouse and human ADAR2. As in the sophisticated data of their previous work (Fig2 in Hideyama et al., 2010), the single-cell-based analysis using microlaserdisection would be the most desirable way to show the precise levels of ADAR2 together with the activity of GluR2 Q/R site-editing.

→ (Response) We thank the reviewer for the valuable comment. In line with the comment, we added Figs. 3C and 4A, and Supporting Information Figs. S3-S5 and S8. We determined the expression level of ADAR2 mRNA using primer pairs that recognize both human and mouse ADAR2 cDNA (Supporting Information Table S2) and found 1.5-fold increase in the level of ADAR2 mRNA compared to wild-type mice (Fig. 4A). The level of ADAR2 protein in the spinal cords and brains did not differ between AR2 mice treated with AAV9-hADAR2 and wild-type mice (Supporting Information Fig. S8). As this reviewer rightly pointed out, we could have definitive information regarding the ADAR2 gene expression if we could determine the expression level of ADAR2 mRNA in individual neurons, but unfortunately we could not amplify Flag mRNA in a single neuron level to differentiate the AAV9-delivered and –non-delivered motor neurons and the level of ADAR2 mRNA expression is below the level of single cell-based analysis.

We have amended the sentences in the Results section (page 7 line 12) as follows.
"The relative abundance of mouse ADAR2 did not significantly differ between the AAV9-injected and saline-injected AR2 mouse spinal cords (Fig 4A) but AAV9-Flag-hADAR2 infection induced 1.5-fold increase in the expression level of total ADAR2 mRNA in the spinal cords (Fig 4A) and brains (Supporting Information Fig S8). Messenger RNA of both hADAR2 and choline acetyltransferase (ChAT) was demonstrated in the spinal cord lysates of AAV9-injected AR2 mice (Fig. 4B), indicating that hADAR2 was delivered to cholinergic AHCs. The editing efficiency at the GluA2 Q/R site was significantly higher in the AAV9-injected AR2 mice than in the control AR2 mice (Fig 4C), although the expression level of ADAR2 protein did not significantly increase in the AAV9-injected mice (Supporting Information Fig S8B, C). "

As for toxicity of additive expression of the exogenous ADAR2 gene and AAV9 infection, we added figures demonstrating lack of glial reactions in the spinal cord of wild-type mice injected with AAV9-GFP (Supporting Information Fig S4) and of AR2 mice injected with AAV9-Flag-hADAR2 (Supporting Information Fig S3, S5). These results were in marked contrast with the marked proliferation of GFAP-positive activated astrocytes and MAC2-postive activated microglial cells in the spinal cord of AR2 mice treated with saline (Supporting Information Fig. S5). A previous report demonstrated that the ADAR2 transgenic mice were phenotypically normal except for moderate obesity (Singh M. et al; 2007, JBC), suggesting the safety of expression of the exogenous ADAR2 gene. Furthermore, we found larger numbers of AHCs in the AAV-treated AR2 mice compared to saline-treated ones with considerable proportions of Flag-ADAR2 positive AHCs (Fig 3C and Supporting Information Fig. S3D), indicating that treatment with AAV9-Flag-ADAR2 rescued AHCs from death in AR2 mice. These results indicate that neither AAV9 infection nor ADAR2 overexpression is toxic to motor and non-motor neurons in mice and AAV9-Flag-ADAR2 treatment is beneficial to AR2 mice.

Also, we have amended the Results and Discussion sections as follows.
"In addition, proliferation of both activated astrocytes showing increased GFAP immunoreactivity and MAC2-positive activated microglial cells was not detected in the spinal cords, including the regions around AAV9-infected neurons of wild-type mice injected with AAV9-GFP and of AR2

mice injected with AAV9-Flag-hADAR2 (Fig 1E, F and Supporting Information Fig S4, S5). There was no significant expression of Flag protein in the peripheral organs, as previously reported (Iwata et al, 2013). These results indicate that the intravenously injected AAV9 vector delivers the *ADAR2* gene to neurons and expression of the delivered *ADAR2* in neurons is not toxic without inducing abnormal glial cell reaction." (Page 5, line 3 from the bottom)
"Furthermore, the expression of the delivered ADAR2 did not induce any adverse effects in neurons or surrounding tissues and prevented the progression of motor dysfunction and neuronal death with restoring ADAR2-mediated RNA editing in AR2 mice (Fig 3, 4 and 5), which provide a mechanistic model of sporadic ALS (Hideyama et al, 2010; Yamashita et al, 2012a). " (Page 9, line 7)

3.Control must be injection of AAV9-mock or AAV9-GFP instead of saline only. The authors stated in Fig 1E-F that proliferation of both activated astrocytes showing increased GFAP immunoreactivity and MAC2-positive activated microglial cells was detected in the anterior horns of AR2 mice injected with AAV9- Flag-hADAR2. This could be due to AAV virus injection. From this point of view, control should be AAV virus injection.

→ (Response) We are sorry to confuse the reviewers and thank the reviewer's careful reading. Due to an unknown reason, the word 'not' was spelled out from the text (page 5 line 1 from the bottom; "--- activated microglial cells was (not) detected ---") As described in the response to the previous comments, injection of AAV9-GFP to wild-type mice did not cause activation of astrocytes or microglia in the spinal cord including the region around the GFP-positive neurons (Supporting Information Fig. S4). Likewise, there was no increase of GFAP or MAC2 immnuoreacitivity around the Flag-positive neurons in AR2 mice treated with AAV9-Flag-ADAR2 despite that there was marked glial proliferation in the spinal cord of AR2 mice treated with saline (Supporting Information Fig S5). Because AAV9 infection and ADAR2 overexpression is not toxic to neurons and ADAR2 expression rescued AHCs from death, we hope the reviewer will accept the use of saline-injected AR2 mice as controls.

4.Two injection groups were applied for the study: 11 animals for the injection at the pre-symptomatic stage and 5 for the injection after the onset. It is confusing since Fig 2A, C, D, and E were for the entire group but only Fig 2B showed the group of the injection after the onset. Fig 2 must be shown separately for each group.

→ (Response) To facilitate reader's understanding, we presented separately the rotarod performance of AR2 mice treated in the pre-symptomatic stage and those treated in the post-symptomatic stage in Supporting Information Fig. S6.

5.The age of mice used in Fig3-5 must be indicated. It is also necessary to specify which treatment group of mice was used, injection before or after the onset.

→ (Response) According to the reviewer's comment, we amended figure legends of Fig.3-5 and added description about the mice used for immunohistochemistry and biochemical analyses in the Materials and Methods section as follows.
"AR2 mice injected with AAV9-hADAR2 vectors before the initiation of motor dysfunction were used for immunohistochemistry and biochemical analyses at 36 weeks of age (n = 5-8). " (Page 15, line 7)

6.Although the age of mice in Fig3 is not specified, the numbers of AHCs/section in Fig3C seem to be apparently discrepant from their previous work (Fig4C in Hideyama et al., 2010). The previous work found the numbers of AHCs per section less than 20 at the age of 2 months. It seems that the age of mice using in Fig3 was near or at the end point that was 9 months.

→ (Response) The difference in the numbers of AHCs per section between this report and our previous one may be due to the difference in the criteria for AHCs. As described in the Materials and Methods section, we counted large anterior horn cells (AHCs) with diameters larger than 20 μm in spinal cord sections stained with TO-PRO-3. On the other hand, we counted AHCs larger than 20 μm in diameter and double-immunopositive for ADAR2 and SMI-32 using DAB as a colour

developer in our previous report (Hideyama et al., 2010). The reviewer will admit that consistent results were obtained using the method adopted in this study

7.The criteria for TDP-43 positive AHCs in Fig 5C are uncertain. The signal quantification is necessary.

→ (Response) There were TDP-43-positive and –negative AHCs (Merge/TO-PRO-3 in Fig. 5A). We designated an AHC as TDP-43-negaive when we could not detect TDP-43 immunofluorescence in the AHC even in the highest sensitivity level of fluorescence microscopy and counted these AHCs as TDP-43(-) in Fig. 5C.

8.In Discussion section (P8, L7-10), the authors stated that "Furthermore, the expression of the delivered ADAR2 prevented the progression of motor dysfunction and neuronal death by normalizing the Ca2+ influx through AMPA receptors and downstream molecular events leading to death, such as calpain activation with resultant mislocalization of TDP-43 in AR2 mice (Fig 3, 4 and 5)". There was no such data of calcium influx or calpain in the manuscript.

→ (Response) In line with the reviewer's appropriate comment, we changed the sentence to describe the summary of the present results as follows,
"Furthermore, the expression of the delivered ADAR2 did not induce any adverse effects in neurons or surrounding tissues and prevented the progression of motor dysfunction and neuronal death with restoring ADAR2-mediated RNA editing in AR2 mice (Fig 3, 4 and 5)," (Page 9, line 7)
In addition, we inserted the mechanistic discussion on how ADAR2 delivery rescued TDP-43 mislocalization to page 10, line 9, as follows.
"Therefore, it is likely that ADAR2 delivery normalized the subcellular localization of TDP-43 in the AHCs by reducing Ca^{2+} influx through AMPA receptors and the resulting calpain activation."

Minor Critiques
1.In P5 L15 what are "remote regions"?

→ (Response) We have amended '' remote regions '' to ''remote regions (ipsilateral hemisphere 2.0-2.5 mm posterior to the injection site)'' (page 5 line 16).

2.Wild-type should be added in Fig 3A, 3C, 4A, and 5C.

→ (Response) We have added data on wild-type mice in Figs. 3A, 3C, 4A and 5C.

3.The description in P6 L16, "apparently, although not significantly, improved" is not scientific.

→ (Response) We have amended "apparently, although not significantly, improved'' to ''AAV9-injected AR2 mice exhibited higher spontaneous locomotor activity than saline-injected AR2 mice, but the difference between the two groups did not reach statistical significance (Fig 2B). The grip power and body weight did not differ between the AAV9-injected and saline-injected AR2 mice (Fig 2C, D).'' (Page 6 line 5 from the bottom) .

4.In P12 L12, "2.14x10^-12" must be "2.14x10^-12 VG/body".

→ (Response) We appreciate your appropriate comment and have amended "2.14x10^-12" to "2.14×10^{12} vg/body".

5.TO-PRO-3 staining was used for detecting the entire AHCs in Fig5. TO-PRO-3 is basically a nuclear staining method. The reason must be mentioned or it would be better to be replaced with a standard neuronal-staining marker.

→ (Response) We thank the reviewer for this comment. Because TO-PRO-3 stains not only the nucleus but the cytoplasm of neurons in the fixed sections of mouse brains and spinal cords, to avoid readers' misunderstanding, we have changed the sentence as follows.
(Figure 5 legends) ''TO-PRO-3 was used as a cell marker''.

© EMBO

6.There are several sentences with no space before parenthesis.

→ (Response) We thank the reviewer for this comment and had amended the sentences.

To Reviewer #2:

Referee #2 (General Remarks):

The authors have determined the ability of ADAR2 expressed from an AAV vector to restore editing function in neurons in a model for ALS, in which endogenous ADAR2 knockout out in motor neurons. If fully validated, this is an important observation.

The critical data appear in Figure 2 in which the authors show that AAV-ADAR2 expression restores rotarod performance in the mouse model. A motor task, and prevents further decline when administered late.

In Figure 1D, Flag-ADAR2 appears to be expressed in neurons that are not CHAT positive. How widespread is the expression?

→ (Response) We thank the reviewer's deep understanding and valuable comments. Because we used the SYN1 promoter, expression of the gene delivered with the AAV9 vector is universal in the central neurons (Iwata et al, 2013 Sci Report). In line with this suggestion, we indicated the expression of Flag-ADAR2 in the spinal cord in Supporting Information Fig. S3A and C.

Figure 4 present the data on the effect of ADAR2 expression in the mouse model on GlyA2 mRNA. It would help to show examples of the original PCR data. The bar graphs are hard to interpret. Specifically, what is the meaning of the statement (Figure 4 legend) that "the relative abundance of hADAR2 mRNA was significantly higher in AR2 mice injected with AAV9-Flag-hADAR2 than in saline injected controls"? There should be absolutely no human ADAR2 mRNA in mouse and if there is a signal, it is possible that the PCR assay is in error. In Figure 4C, there is only a modest change in editing efficiency with AAV expression of ADAR2. How can such a small change account for the big recovery of rotarod function seen in Figure 2?

→ (Response) We quantified ADAR2 mRNA using a lightcycler 480 (Roche Instruments), and the results were presented as computerized ones using internal standards for reference (Sawada et al, 2009; Yamashita et al, 2012c). Therefore, we could not provide "original PCR data".

As the reviewer rightly pointed out, no signal for human ADAR2 mRNA should be detectable in the samples from saline-injected AR2. Actually, however, very low levels of signal could not be eliminated from reasons residing in quantitative PCR system. To facilitate the readers' understanding, we added the data illustrating the upregulation of total ADAR2 (using primer pairs that recognize both human and mouse ADAR2 cDNA) to Fig. 4A and amended the figure legend as follows.

"The relative abundance of mouse ADAR2 mRNA did not significantly differ between AR2 mice injected with AAV9-Flag-hADAR2 (AAV) and those injected with saline (Saline; n=5 for each group). hADAR2 mRNA was expressed at a significant level in AAV. The relative abundance of total (human and mouse) ADAR2 mRNA was 1.5-fold higher in AAV (n=4) than in Saline (n=4). **$p < 0.01$ (Mann–Whitney U-test). All error bars represent the s.e.m."

As for the results presented in Fig. 4C, we would like attract the reviewer's attention to the fact that the number of remaining AHCs is about 30% higher in the AAV group than in the saline group (Fig. 3C and Supplementary Information Fig. S3D). Because the majority of AHCs that expressed unedited GluA2 had already disappeared at the age of examination, editing efficiency was compared between the remaining AHCs that express ADAR2. Therefore, apparently small decrease in the expression level of unedited GluA2 may result from the significant increase in the expression level of ADAR2 with a big difference in neuronal functions. Larger number and a higher ADAR2 activity in the remaining AHCs of AAV-treated AR2 mice likely result in better behavioural and morphological changes than in saline-treated AR2 mice.

To Reviewer #3:

The authors consider that the downregulation of adenosine deaminase acting on RNA1 (ADAR2) is a general pathogenic mechanism in Amyotrophic Lateral Sclerosis. TDP43 accumulation in the cytoplasm is, in their view, related to the expression of abnormal Ca2+-permeable AMPA receptor through activation of calpain. The hypothesis is interesting but has been developed and tested only by the research group of the authors and awaits confirmation from other teams.

→ (Response) We appreciate your comment and confirmation study of our hypothesis by other teams is highly welcome.

Referee #3 (General Remarks):

The authors hypothesize that the downregulation of adenosine deaminase acting on RNA1 (ADAR2) is a general pathogenic mechanism in Amyotrophic Lateral Sclerosis (ALS). TDP43 accumulation in the cytoplasm is, in their view, related to the expression of abnormal Ca2+-permeable AMPA receptor through activation of calpain. To test further their hypothesis, they treated ADAR2 conditional KO mice which develop a motor deficit (AR2 mice)with an AAV9 vector containing human ADAR2 gene. The treatment prevented other neuronal loss and the loss of axons.
The paper is clear. I would however recommend providing larger and higher quality histological pictures. Fig 1, E and F are barely visible.

→ (Response) We thank the reviewer for the comment. We have added better histological pictures in Supporting Information Fig S5.

In fig 2, a bar should indicate that the significant difference was between the saline treated and the AAV treated animal.

→ (Response) We thank the reviewer for the comment and have amended Fig 2 and figure legend to indicate that the difference was between the saline- and AAV-treated groups.

In fig 3 A, no histological difference is visible between saline and AAV treated animals. The density of myelin fibres appears to remain very high in the AR2 mice. A better view of the histological sections should demonstrate the myelin loss.

→ (Response) We thank the reviewer for the comment and have added additional examples that demonstrate axonal degeneration in the ventral roots of the saline-treated but not of the AAV-treated AR2 mice in Supporting Information Fig S7 to facilitate readers' understanding.

In fig 4,C I believe that the n used to compute the SEM is the number of samples (4 samples by mouse for 8 and 7 individuals per group). This explains the very low SEM and the statistical significance between the groups despite the small difference between the means. I believe the correct methodology would be to compute mean per individual, to calculate a SEM using these (mean) individual values with n=8 and 7 respectively. I am not convinced that the difference would remain significant.

→ (Response) We also tested the difference between the groups and found the difference was significant (Fig. 4C legend). As described in the response to the previous comments, the AHCs expressing abundant unedited GluA2 had disappeared at the time of examination and we compared remaining AHCs that express ADAR2 between the two groups. An increase of editing efficiency in the AHCs in the AAV-treated group reflects the increase in the ADAR2 level resulting from additional expression of the delivered *ADAR2* gene.

Fig 5,A TDP43 IHC is negative in the nuclei but there is apparently no labelling in the cytoplasm in the saline treated animals. Was this expected? Is there no inclusion in the cytoplasm as in ALS? Could the authors provide a better view of the pathological and rescued anterior horn cells (larger picture, higher magnification)?

→ (Response) We have added Supporting Information Fig. S9 to show the TDP-43 pathology in AR2 mice more clearly and how it was rescued by AAV-ADAR2. As the reviewer predicted, a few motor neurons exhibit multiple TDP-43-positive aggregates in the cytoplasm (Supporting Information Fig. S9B). These neurons lack nuclear TDP-43 immunoreactivity as well, displaying abnormal view similar to ALS motor neurons.
Also, we have amended the results section (Page 7 line 7 from the bottom) as follows.

"Consistent with the effective prevention of the death of AHCs, loss or mislocalization of TDP-43 in the AHCs of saline-treated AR2 mice was rescued in AAV9-injected AR2 mice, and Flag-expressing AHCs exhibited predominantly nuclear TDP-43 localization (Fig 5 and Supporting Information Fig S9)."

We are looking forward to hearing favourable decision on this revised manuscript.

Additional Editorial Correspondence 17 August 2013

Thank you for resubmitting your manuscript for our consideration. As I would like to make an informed decision on your manuscript, I need clarification from your part before to proceed.

I have now received the two sets of reviews I asked for and I am afraid that referee 1 is still not satisfied (please see below) and remains concerned about the similar ADAR2 mRNA expression reported between saline control and WT (Figure 4A). We agree with the referee that this result is indeed confusing as a difference needs to be shown to validate that the gene therapy approach using a single systemic delivery fully works.

As such, I would appreciate if you could let me know whether you would be prepared to convincingly address this issue for the paper to be accepted and how you would propose to do so (using the proposed experiment or something else to same effect).

Please see below the report provided by referee 1:

The authors added some experiments to answer the critics raised by the reviewer and the manuscript was improved partially. Nevertheless, there were still several issues with regard to the validation of experimental models, which made the entire manuscript unconvincing. Therefore the reviewer recommends the authors to send the manuscript to a more specific journal after careful revision.

1. It was appreciable that the authors used the primers that recognize both mouse and human ADAR2 mRNA for gene expression analysis in Figure 4A. However, there was no difference of total ADAR2 mRNA expression between saline control and WT (Figure 4A, right), which means there was no reduction of ADAR2 mRNA levels in saline control of ADAR2 KO mice compared to WT control. It is quite confusing for readers and two possibilities can explain the results; there was not enough reduction of ADAR2 mRNA in the ADAR2 KO mice used in the study for some reason, or the methods/technology used in Fig 4 were not good enough to confirm endogenous/exogenous gene expression levels of ADAR2. I guess it was because the entire tissue homogenization process masked motor neuron-specific knock-out of ADAR2 at the ventral horn as the protein levels of ADAR2 was also comparable in Fig. S8. Since the virtue of this model is the whole body administration of AAV-ADAR2 by a "single intravenous injection", the authors should validate expression of delivered gene in the targeted neurons precisely. Therefore, in the first review comments the authors were recommended to confirm ADAR2 mRNA levels by single cell capture-based method which had been used in their previous study (Hideyama et al., 2010) if immunohistochemistry was not applicable. Unfortunately, the authors

just mentioned "level of ADAR2 mRNA expression is below the level of single cell-based analysis", although they successfully showed the data of ADAR2 mRNA changes in single cells captured by laser micro dissection (Fig2 in Hideyama et al., 2010).

2. The way of TDP-43 positive AHCs measurement was still subjective and not acceptable. They should use objective criteria for it, such as certain cut-off signal intensity.

Additional Author Correspondence 20 August 2013

Thank you for the e-mail regarding our submitted manuscript. Below I am writing the response to the comments from Reviewer 1.

The critics raised by the reviewer 1 seem to be three-fold.

1) The reason why there is no difference in the ADAR2 expression level between the wild-type mice and saline control AR2 mice.
2) Lack of evidence of expression of AAV-delivered ADAR2 to motor neurons after single systemic injection of AAV9 vector.
3) Validation of TDP-43 immunohistochemical positivity.

Response:
1) As we have responded to the previous comments, qPCR for ADAR2 in a single neuron level is not possible. We tried to validate the qPCR for ADADR2 in the mouse spinal cord and laser-captured motor neurons, but found that spinal cord tissue larger than 1 mm-thick axial slice was necessary for reliable results. This means that thousands or more of motor neurons are necessary. We tried to demonstrate the reduction in ADAR2 mRNA expression in AR2 mice compared to wild-type mice by qPCR but were unsuccessful. This may be due to the fact that less than 50% of reduction in the number of motor neurons may be masked in the remaining motor and non-motor neurons in the ventral horns, as reviewer 1 rightly predicted.
Although the reviewer 1 stated that we previously reported the results of qPCR for ADAR2 in a single cell level in Fig. 2 of Hideyama et al. 2010 J Neurosci, what we presented there was the ordinary PCR for ADAR2 on pooled mouse motor neuron lysates but not the qPCR data. We presented qPCR for ADAR2 on pooled human motor neuron lysates in Fig. 2 of Hideyama et al., 2012 Neurobiol Dis, but the expression level of ADAR2 mRNA was more than one-order higher in human motor neurons than in mouse motor neurons.Another thing one should take into account was that the majority of ADAR2-lacking motor neurons had been already disappeared and the majority of remaining motor neurons were those expressing ADAR2 in the control AR2 mice at the age of examination. Therefore, it would be technically difficult to differentiate 10-15% difference of ADAR2 mRNA expression level even in the pooled motor neurons between the saline control AR2 mice and wild-type mice as stated above. We cannot tell the difference in the expression level of ADAR2 mRNA by means of ordinary PCR. There may be some misunderstanding.
We presented the significant reduction in the number of motor neurons and axons in the ventral root of saline control AR2 mice compared to wild-type mice in Fig. 3, which was consistent with the results in the original report (Hideyama et al., 2010 J Neurosci) indicating that lack of ADAR2 induced death in the motor neurons in the AR2 mice.
2) To fill the defect of quantitative data on ADAR2 expression, we presented the GluA2 Q/R site-editing in a single motor neuron level in Fig. 4C. Because GluA2 Q/R site-editing is specifically catalyzed by ADAR2, an increase in the editing efficiency in the AAV group compared to the saline control AR2 mice indicate that exogenous ADAR2 is successfully delivered to and expressed in the motor neurons. This method for ADAR2 activity in a single cell was the same as we adopted in Fig. 2A of Hideyama et al, 2010, J Neurosci. We also presented that the AAV vector was delivered to motor neurons by demonstrating Flag expression in ChAT-positive large neurons in the anterior horn of the spinal cord in Fig. 1D and more ADAR2-positive AHCs were remained in AAV-treated AR2 mice than in the saline control AR2 mice in Fig. S3D. We believe that these data sufficiently indicate that exogenously delivered ADAR2 was successfully expressed in the motor neurons by

single systemic injection of the AAV vector.
3) As for the TDP-43 immunohistochemistry, in line with the reviewer 1's comment, we adopted the criteria that the positivity is above 3-fold of background. For reviewer 1's convenience, the results of quantification are attached and the method used is indicated in the Mariterials and Methods section. The results did not change after adopting the criteria.

We believe we could appropriately respond to all the concerns raised by reviewer 1's and the editors.

3rd Editorial Decision 20 August 2013

Thank you for the submission of your revised manuscript to EMBO Molecular Medicine and your further correspondence.

We have now received the enclosed reports from the referees that were asked to re-assess it. As you will see from the pasted comments below and following our recent e-mail exchange, I am happy to let you know that the reviewers are now supportive and that we will be able to accept your manuscript pending the following final amendments:

- Please modify the final text of the manuscript to remove any blue text.

- In light of the document provided to referee 1, would you agree to incorporate this data within the supplemental information and therefore change the text accordingly? Similarly, as you may know we will publish the referees' reports along with your reply to their comments online and will incorporate our latest discussion (see below). Please do let us know immediately if you would object.

Please submit your revised manuscript as soon as possible.

I look forward to hearing from you soon.

***** Reviewer's comments *****

Referee #1 (General Remarks):

The authors added some experiments to answer the critics raised by the reviewer and the manuscript was improved partially. Nevertheless, there were still several issues with regard to the validation of experimental models, which made the entire manuscript unconvincing. Therefore the reviewer recommends the authors to send the manuscript for more specific journal after careful revision.

1. It was appreciable that the authors used the primers that recognize both mouse and human ADAR2 mRNA for gene expression analysis in Figure 4A. However, there was no difference of total ADAR2 mRNA expression between saline control and WT (Figure 4A, right), which means there was no reduction of ADAR2 mRNA levels in saline control of ADAR2 KO mice compared to WT control. It is quite confusing for readers and two possibilities can explain the results; there was not enough reduction of ADAR2 mRNA in the ADAR2 KO mice used in the study for some reason, or the methods/technology used in Fig 4 were not good enough to confirm endogenous/exogenous gene expression levels of ADAR2. I guess it was because the entire tissue homogenization process masked motor neuron-specific knock-out of ADAR2 at the ventral horn as the protein levels of ADAR2 was also comparable in Fig. S8. Since the virtue of this model is the whole body administration of AAV-ADAR2 by a "single intravenous injection", the authors should validate expression of delivered gene in the targeted neurons precisely. Therefore, in the first review comments the authors was recommended to confirm ADAR2 mRNA levels by single cell capture-based method which had been used in their previous study (Hideyama et al., 2010) if immunohistochemistry was not applicable. Unfortunately, the authors just mentioned "level of ADAR2 mRNA expression is below the level of single cell-based analysis", although they

successfully showed the data of ADAR2 mRNA changes in single cells captured by laser micro dissection (Fig2 in Hideyama et al., 2010).

2. The way of TDP-43 positive AHCs measurement was still subjective and not acceptable. They should use objective criteria for it, such as certain cut-off signal intensity.

Referee #2 (Comments on Novelty/Model System):

The model system is adequate and was developed by the authors.

Referee #2 (General Remarks):

The authors have addressed my concerns. The medical question posed here is highly significant although it has been pursued almost exclusively by the authors. The data support their conclusions although there is clearly substantial scope for further investigation of this model and the underlying medical problem.

2nd Revision - authors' response 23 August 2013

We are grateful to have heard your favourable decision with appropriate comments of the reviewers. We carefully revised our manuscript, figures and supplementary figures and tables in line with reviewers' comments as described bellow. In addition, we made some changes for the refinement of style. The total number of words in the abstract is 173 and the total number of words in the main text is 7893. The revised manuscript is with 29 references, 5 figures, 10 Supporting Information figures and 2 Supporting Information tables.

In line with the comments from editors and reviewers, we have provided additional data to present the results more clearly with sufficient discussion in the revised manuscript. Below, we provide responses (beginning at each arrow) that specifically address each of the reviewers' comments, which are italicized.

To Reviewer #1:

Referee #1 (General Remarks):

The authors added some experiments to answer the critics raised by the reviewer and the manuscript was improved partially. Nevertheless, there were still several issues with regard to the validation of experimental models, which made the entire manuscript unconvincing. Therefore the reviewer recommends the authors to send the manuscript for more specific journal after careful revision.

1. It was appreciable that the authors used the primers that recognize both mouse and human ADAR2 mRNA for gene expression analysis in Figure 4A. However, there was no difference of total ADAR2 mRNA expression between saline control and WT (Figure 4A, right), which means there was no reduction of ADAR2 mRNA levels in saline control of ADAR2 KO mice compared to WT control. It is quite confusing for readers and two possibilities can explain the results; there was not enough reduction of ADAR2 mRNA in the ADAR2 KO mice used in the study for some reason, or the methods/technology used in Fig 4 were not good enough to confirm endogenous/exogenous gene expression levels of ADAR2. I guess it was because the entire tissue homogenization process masked motor neuron-specific knock-out of ADAR2 at the ventral horn as the protein levels of ADAR2 was also comparable in Fig. S8. Since the virtue of this model is the whole body administration of AAV-ADAR2 by a "single intravenous injection", the authors should validate expression of delivered gene in the targeted neurons precisely. Therefore, in the first review comments the authors was recommended to confirm ADAR2 mRNA levels by single cell capture-based method which had been

used in their previous study (Hideyama et al., 2010) if immunohistochemistry was not applicable. Unfortunately, the authors just mentioned, "level of ADAR2 mRNA expression is below the level of single cell-based analysis", although they successfully showed the data of ADAR2 mRNA changes in single cells captured by laser micro dissection (Fig2 in Hideyama et al., 2010).

→ (Response) The points of critics raised by the reviewer 1 could be summarized as the followings.
1) The reason why there is no difference in the ADAR2 expression level between the wild-type mice and saline control AR2 mice.
2) Lack of evidence of expression of AAV-delivered ADAR2 to motor neurons after single systemic injection of AAV9 vector.

1) As we have responded to the reviewer's comments for the original manuscript, qPCR for ADAR2 in a single neuron level is not possible. We tried to validate the qPCR for ADADR2 in the mouse spinal cord and laser-captured motor neurons, but found that spinal cord tissue larger than 1 mm-thick axial slice was necessary for reliable results. This means that thousands or more of motor neurons are necessary. We tried to demonstrate the reduction in ADAR2 mRNA expression in AR2 mice compared to wild-type mice by qPCR but were unsuccessful. This may be due to the fact that less than 50% of reduction in the number of motor neurons may be masked in the remaining motor and non-motor neurons in the ventral horns, as reviewer 1 rightly predicted.

Although the reviewer 1 stated that we previously reported the results of qPCR for ADAR2 in a single cell level in Fig. 2 of Hideyama et al. 2010 (J Neurosci 30:11917-25), what we presented there was the ordinary PCR for ADAR2 on pooled mouse motor neuron lysates but not the qPCR data. We presented qPCR for ADAR2 on pooled human motor neuron lysates in Fig. 2 of Hideyama et al., 2012 (Neurobiol Dis 45:1121-28), but the expression level of ADAR2 mRNA was more than one-order higher in human motor neurons than in mouse motor neurons. Another thing one should take into account was that the majority of ADAR2-lacking motor neurons had been already disappeared and the majority of remaining motor neurons were those expressing ADAR2 in the control AR2 mice at the age of examination. Therefore, it would be technically difficult to differentiate 10-15% difference of ADAR2 mRNA expression level even in the pooled motor neurons between the saline control AR2 mice and wild-type mice as stated above. We cannot tell the difference in the expression level of ADAR2 mRNA by means of ordinary PCR. There may be some misunderstanding.

We presented the significant reduction in the number of motor neurons and axons in the ventral root of saline control AR2 mice compared to wild-type mice in Fig. 3, which was consistent with the results in the original report (Hideyama et al., 2010 J Neurosci) indicating that lack of ADAR2 induced death in the motor neurons in the AR2 mice.

2) To fill the defect of quantitative data on ADAR2 expression, we presented the GluA2 Q/R site-editing in a single motor neuron level in Fig. 4C. Because GluA2 Q/R site-editing is specifically catalysed by ADAR2, an increase in the editing efficiency in the AAV group compared to the saline control AR2 mice indicate that exogenous ADAR2 is successfully delivered to and expressed in the motor neurons. This method for ADAR2 activity in a single cell was the same as we adopted in Fig. 2A of Hideyama et al, 2010, J Neurosci. We also presented that the AAV vector was delivered to motor neurons by demonstrating Flag expression in ChAT-positive large neurons in the anterior horn of the spinal cord in Fig. 1D and more ADAR2-positive AHCs were remained in AAV-treated AR2 mice than in the saline control AR2 mice in Supporting Information Fig. S3D. We believe that these data sufficiently indicate that exogenously delivered ADAR2 was successfully expressed in the motor neurons by single systemic injection of the AAV vector.

To facilitate readers' understanding, we amended the sentences in the Results and Discussion sections as follows, and added relevant figure numbers in the Discussion section;

"Because rescue of death of AHCs likely results from restoration of ADAR2 activity in the motor neurons of AR2 mice (Hideyama et al, 2010), we next investigated whether the expression and activity of ADAR2 were increased in the motor neurons after systemic injection of AAV9-hADAR2." (Page 7 line 12)

"<u>Messenger RNA of both hADAR2 and choline acetyltransferase (ChAT) was demonstrated in the spinal cord lysates of AAV9-injected AR2 mice (Fig. 4B) and</u> the editing efficiency at the GluA2 Q/R site was significantly higher in the remaining motor neurons of AAV9-injected AR2 mice than in those of the control AR2 mice<u> (Fig 4C). These results indicated that hADAR2 was delivered to and functioned in motor neurons. However, ADAR2 protein level did not significantly differ among AAV9-injected AR2 mice, saline-injected AR2 mice and wild-type mice (Supporting Information Fig S8B, C)</u>. <u>The failure to detect the difference despite of the difference in the ADAR2 activity was</u>

presumably due to the fact that a modest increase in the ADAR2 expression level with preservation of death of 10-20% of motor neurons in AAV-treated AR2 mice may be masked in ADAR2 expressed in the remaining motor and non-motor neurons and other cells in the anterior horn." (Page 7, line 19)
"Furthermore, the expression of the delivered ADAR2 prevented the progression of motor dysfunction and neuronal death with restoring ADAR2-mediated RNA editing (Figs 2, 3, 4 and Supporting Information Fig S7) without inducing any adverse effects in neurons or surrounding tissues (Fig 1 and Supporting Information Figs S4, S5)" (Page 9, line 8).

2. The way of TDP-43 positive AHCs measurement was still subjective and not acceptable. They should use objective criteria for it, such as certain cut-off signal intensity.

→ (Response) We thank the reviewer for the comment. According to reviewer's comment, we added Supporting information Fig. S10. As for the TDP-43 immunohistochemistry, we adopted the criteria that the positivity is above 3-fold of background. For reviewer 1's convenience, the results of quantification are attached and the method used is indicated in the Materials and Methods section. The results did not change after adopting the criteria.
We have added the sentences in the Materials and Methods section as "The signal intensity of TDP-43 was examined using Image J software. TDP-43-positive AHCs were counted when the signal intensity was more than 3-fold higher than the background". (Page 19 line 6 from the bottom).

To Reviewer #2:

Referee #2 (Comments on Novelty/Model System):

The model system is adequate and was developed by the authors.

Referee #2 (Remarks):

The authors have addressed my concerns. The medical question posed here is highly significant although it has been pursued almost exclusively by the authors. The data support their conclusions although there is clearly substantial scope for further investigation of this model and the underlying medical problem.

→ (Response) We thank the reviewer's deep understanding and valuable comments.

We are looking forward to hearing favourable decision on this revised manuscript.
