# Supplementary material for: Rescue of amyotrophic lateral sclerosis phenotype in a mouse model by intravenous AAV9-ADAR2 delivery to motor neurons
Source: EMBO Mol Med. 2013 Sep 24;5(11):1710–9. doi: 10.1002/emmm.201302935 (PMC3840487; doi:10.1002/emmm.201302935)
Supplement: Supplementary file 2 [file emmm0005-1710-SD2.pdf]

Rescue of amyotrophic lateral sclerosis phenotype in a mouse model by intravenous AAV9-*ADAR2* delivery to motor neurons

Takenari Yamashita, Hui Lin Chai, Sayaka Teramoto, Shoji Tsuji, Kuniko Shimazaki, Shin-ichi Muramatsu & Shin Kwak

SUPPORTING INFORMATIONS

Supporting Information Figure S1. Viral transduction of the AAV9-SynI vector in the mouse.

Supporting Information Figure S2. Expression of AAV-delivered ADAR2 in Neuro2a cells and wild-type mouse brains.

Supporting Information Figure S3. Expression of AAV9-Flag-hADAR2 in the AR2 mouse spinal cord after intravenous injection.

Supporting Information Figure S4. GFAP and MAC2 immunoreactivity in the spinal cords of wild-type mice injected with AAV9-GFP.

Supporting Information Figure S5. GFAP and MAC2 immunoreactivity in the spinal cord of AAV-injected AR2 mice.

Supporting Information Figure S6. Rotarod performance of AAV-injected AR2 mice.

Supporting Information Figure S7. Magnified view of the ventral roots of the fifth lumber segment (L5).

Supporting Information Figure S8. Effects on the expression of ADAR2 in the brain and spinal cord.

Supporting Information Figure S9. Rescue of TDP-43 in motor neurons.

Supporting Information Figure S10. The signal intensity of TDP-43-positive and -negative AHCs.

Supporting Information Table S1. Primer sequences.

Supporting Information Table S2. TaqMan probes used for quantitative polymerase chain reaction.

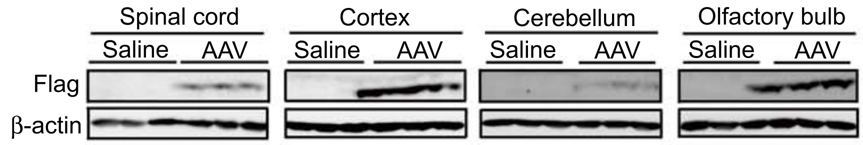

Supporting Information Figure S1. Viral transduction of the AAV9-SynI vector in the mouse.

Western blot analyses using an anti-Flag-tag antibody showed a widespread distribution of the AAV9-SynI vector in the central nervous system, including the spinal cord, cortex, cerebellum and olfactory bulb of AR2 mice intravenously injected with AAV9-Flag-hADAR2 (AAV). Saline; AR2 mice treated with saline.

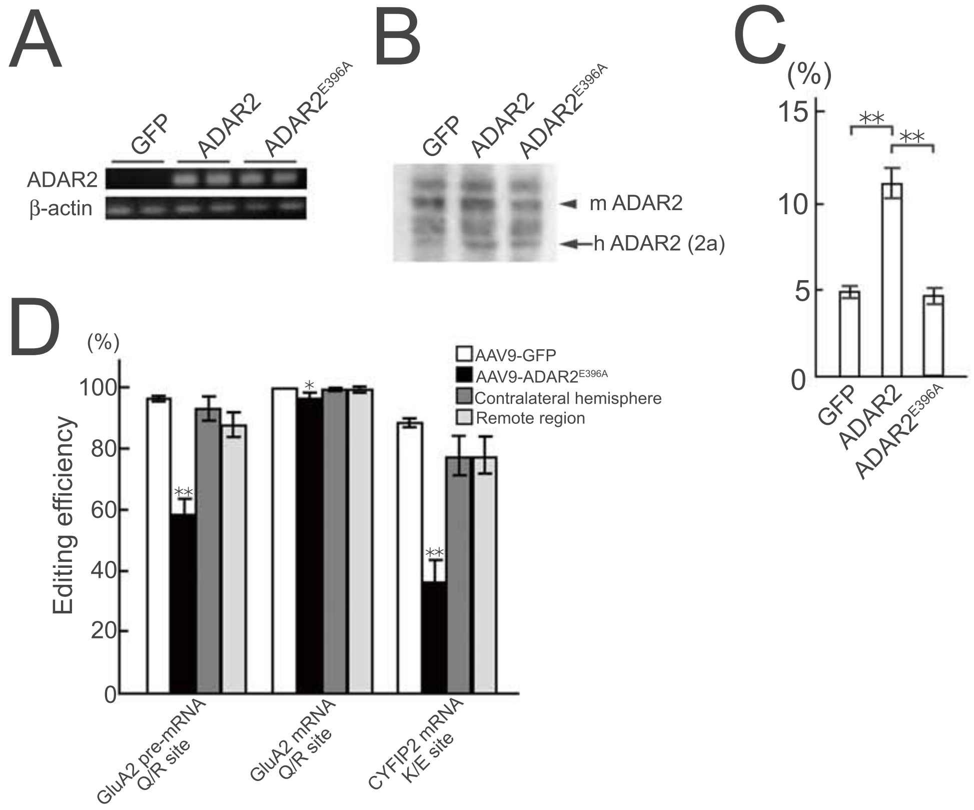

Supporting Information Figure S2. Expression of AAV-delivered ADAR2 in Neuro2a cells and wild-type mouse brains.

A. B. RT-PCR (**A**) and Western blot analyses (**B**) of ADAR2 demonstrated that hADAR2 was expressed in Neuro2a cells infected with AAV9-hADAR2 or AAV9-hADAR2^{E396A}.

C. The editing efficiency at the CYFIP2 mRNA K/E site was significantly higher in Neuro2a cells infected with AAV9-hADAR2 compared with those infected with AAV9-GFP- or AAV9-hADAR2^{E396A} (mean ± s.e.m.; n=6 for each group). **P<0.01 (Mann -Whitney U-test).

D. Editing efficiencies at the Q/R site of GluA2 pre-mRNA and the K/E site of CYFIP2 mRNA in the brains of wild-type mice injected with AAV9-hADAR2^{E396A} in the cerebral cortex. Editing efficiencies were significantly decreased at the injection site, but not in the remote ipsilateral (2.0-2.5 mm posterior to the injection site) or contralateral hemisphere. *P<0.05, ***P<0.0001 (Mann -Whitney U-test,). The editing efficiencies at these sites were not changed in the mouse brain area injected with AAV9-GFP. All error bars represent the s.e.m., n = 4 - 12.

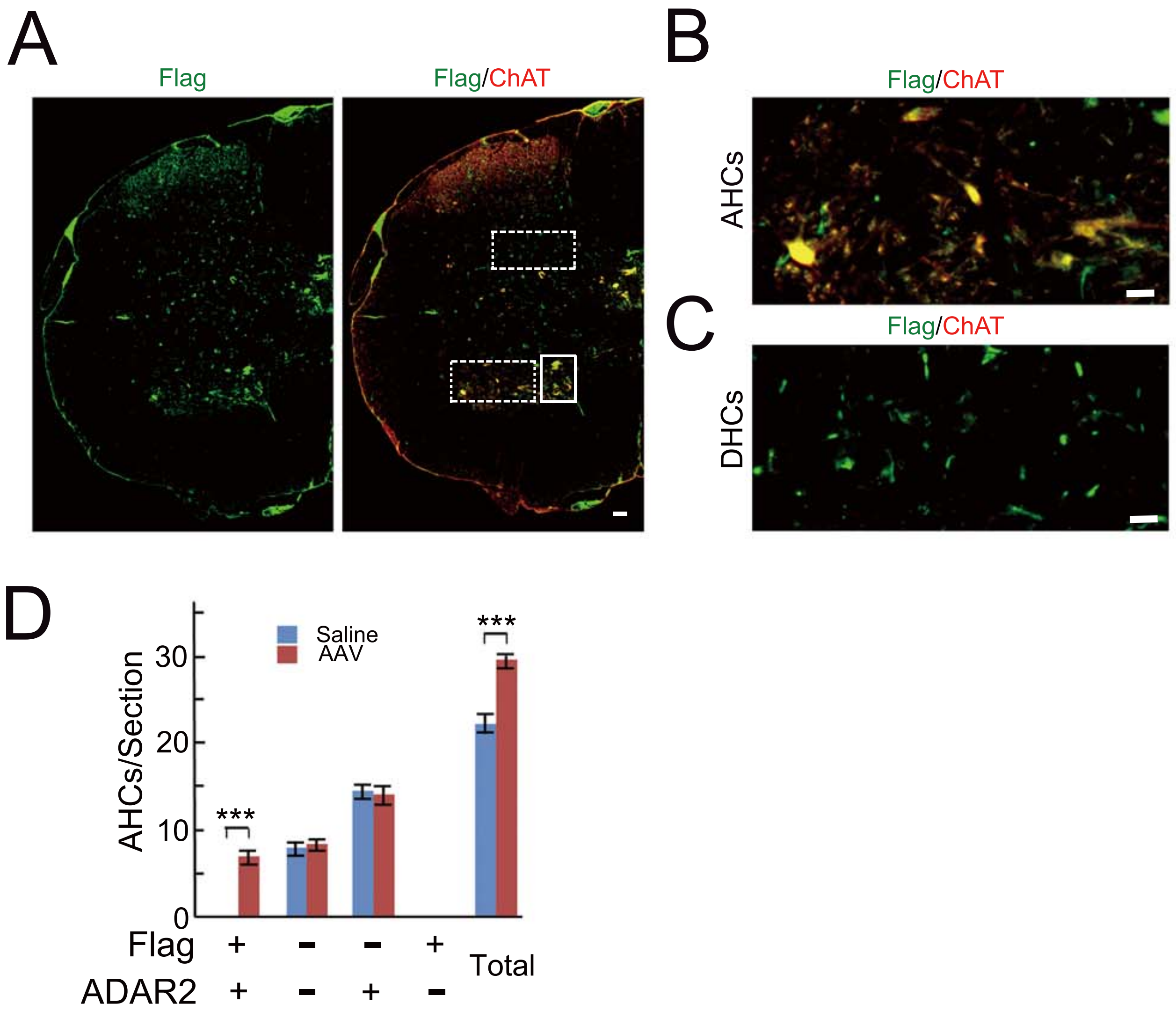

Supporting Information Figure S3. Expression of AAV9-Flag-hADAR2 in the AR2 mouse spinal cord after intravenous injection.

A. Immunohistochemistry for Flag (green) and ChAT (red) in the spinal cords of an AR2 mouse intravenously injected with AAV9-Flag-hADAR2. The boxed areas represent the area for high power magnification image of Figure 1D. Scale bar, 50 μm.

B. C. Magnified view of the dotted line areas in **A**. Both ChAT-positive anterior horn cells (AHCs) (**B**) and ChAT-negative dorsal horn cells (DHCs) (**C**) exhibit Flag immunoreactivity. Scale bars, 20 μm.

D. Numbers of AHCs classified by immunoreactivity to Flag and ADAR2 in AR2 mice intravenously injected with AAR9-Flag-hADAR2 or saline. AR2 mice ($n = 4$ for each group) were examined at 33-36 weeks of age, ***$p < 0.001$ (Student's t-test). All error bars represent the s.e.m.

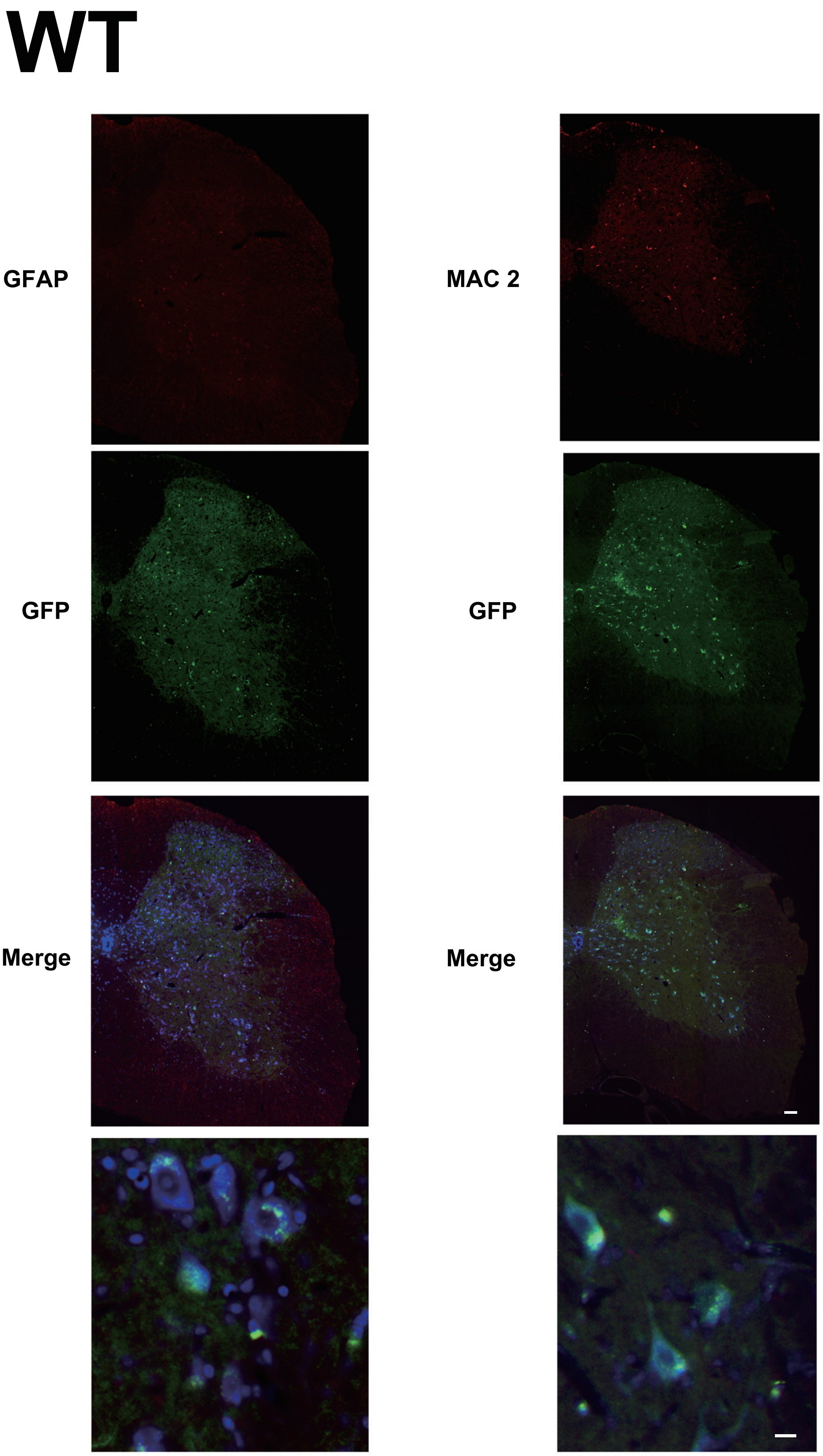

Supporting Information Figure S4. GFAP and MAC2 immunoreactivity in the spinal cords of wild-type mice injected with AAV9-GFP.

Axial section of the spinal cord of wild type mice (21 weeks of age) 11 weeks after the intravenous injection with AAV9-GFP (7.2 x 10^{11} vg/body). There was little or virtually no increase of GFAP and MAC2 immunoreactivity in the spinal cord. There was no GFAP-positive astrocytes or MAC2-positive microglia around the GFP-positive neurons (bottom). Scale bars, 50 µm (upper panels) and 20 µm (bottom panels).

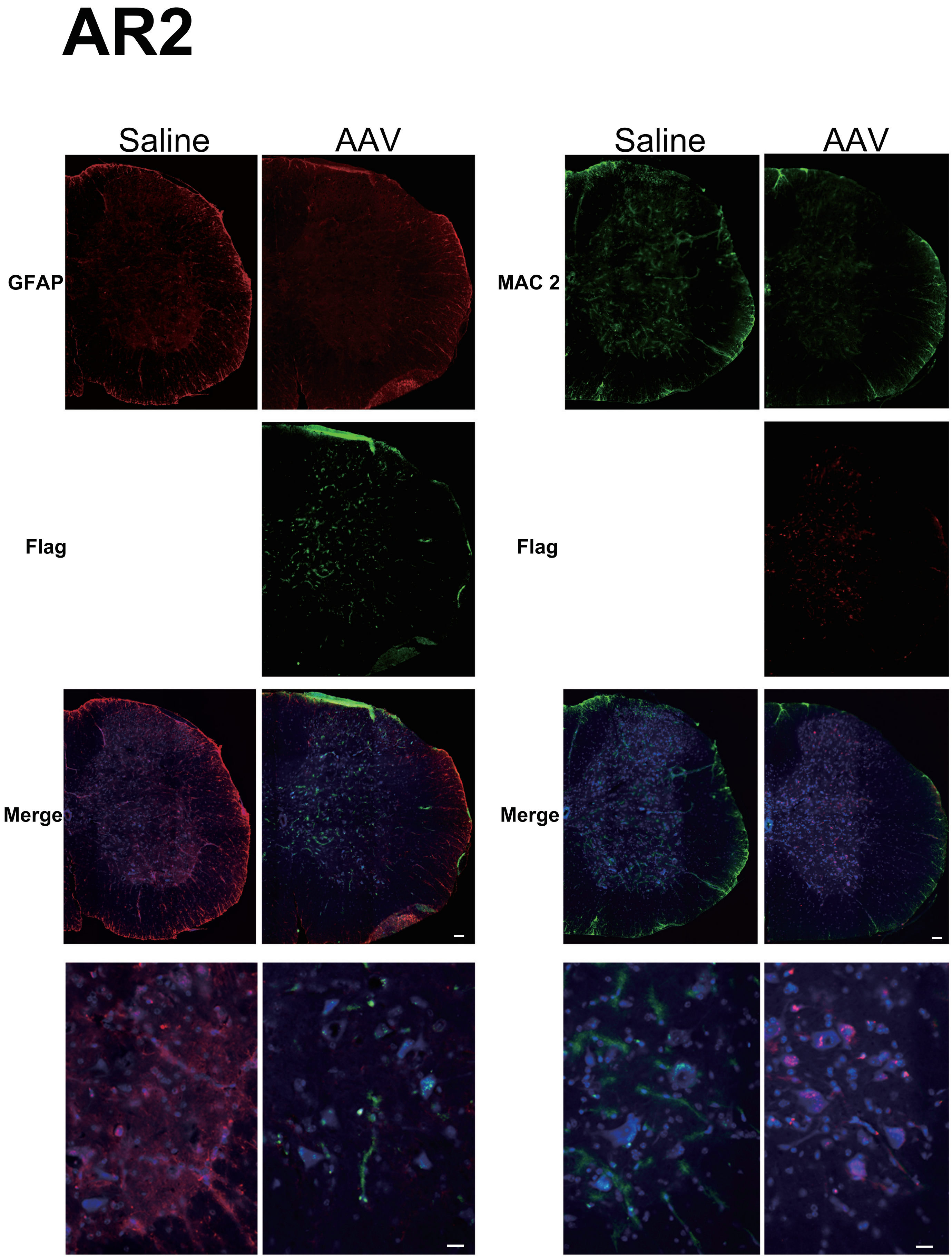

Supporting Information Figure S5. GFAP and MAC2 immunoreactivity in the spinal cord of AAV-injected AR2 mice.

In contrast to the intense GFAP and MAC2 immunoreactivity in the spinal anterior horns (AH) of the saline-treated AR2 mice (Saline), there was virtually no increase of the immunoreactivity in the AH of AR2 mice intravenously injected with AAV9-Flag-hADAR2 (AAV). Scale bars, 50 μm (upper panels) and 20 μm (bottom panels).

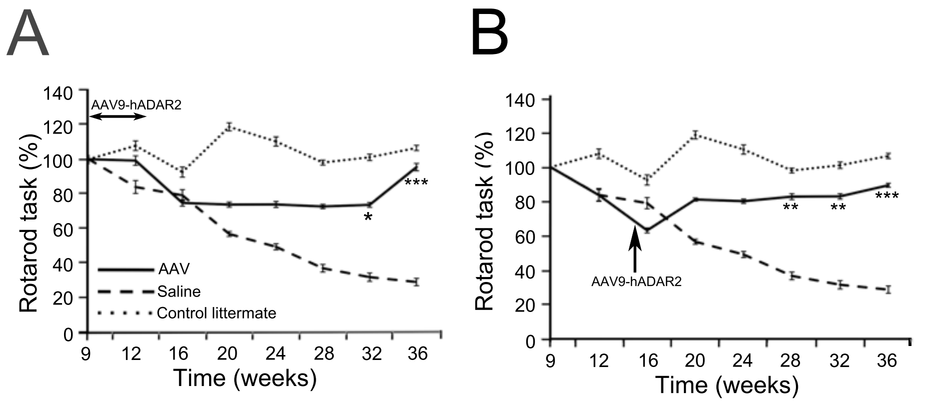

Supporting Information Figure S6. Rotarod performance of AAV-injected AR2 mice.
A. Rotarod performance was significantly better in AR2 mice intravenously injected with AAV9-Flag-hADAR2 at presymptomatic stages (n=11; AAV) than in saline-treated AR2 mice (n=13; Saline) (Student's t-test against Saline group, *$p < 0.05$, ***$p < 0.0001$). All error bars represent the s.e.m.
B. Injection of the AAV vector into AR2 mice that already exhibited declined rotarod performance prevented the progression of motor dysfunction (n=5; AAV; Student's t-test against Saline group, **$p < 0.01$, ***$p < 0.0001$). All error bars represent the s.e.m.

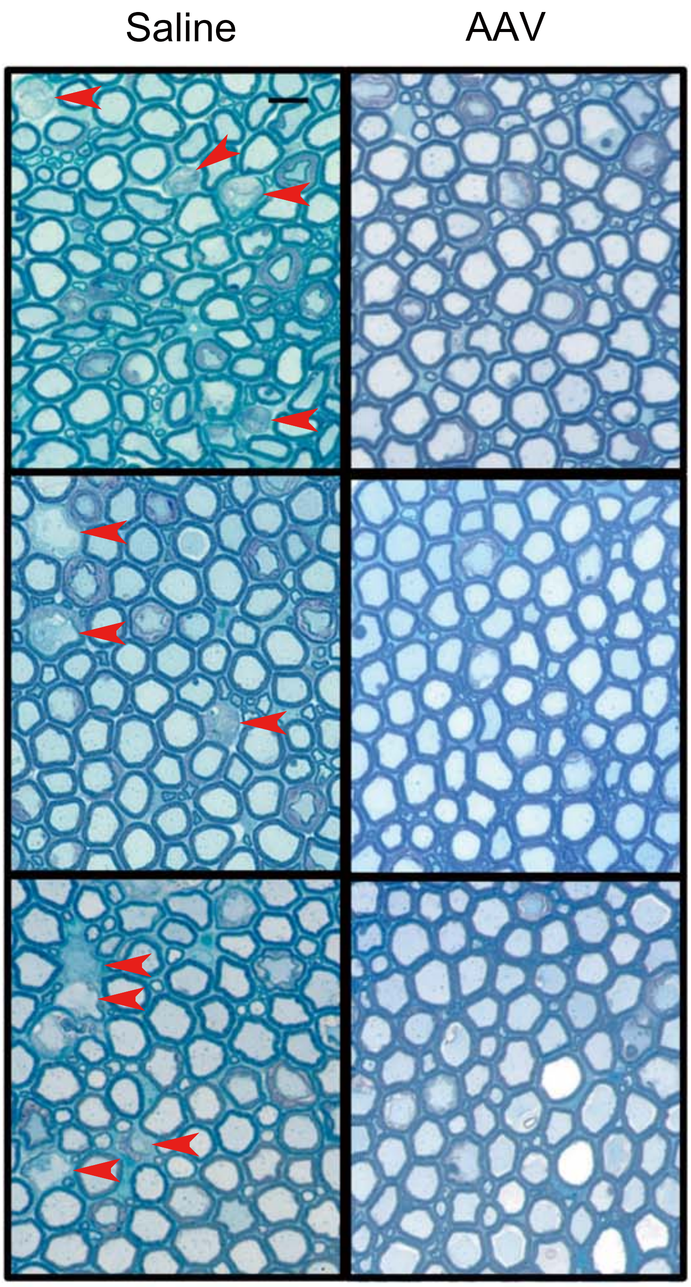

Supporting Information Figure S7. Magnified view of the ventral roots of the fifth lumber segment (L5)

Each panel is the ventral root of either saline-injected (Saline, n=3) or AAV9-Flag-hADAR2-injected AR2 mice (AAV, n=3). Arrowheads indicate axonal degeneration. Scale bar: 10 µm.

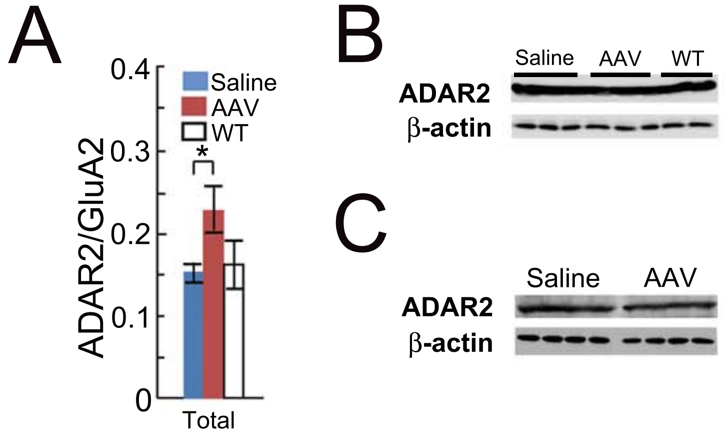

Supporting Information Figure S8. Effects on the expression of ADAR2 in the brain and spinal cord.

A. The relative abundance of hADAR2 mRNA was significantly higher in the brains of AR2 mice injected with AAV9-Flag-hADAR2 (AAV) (n=3) than in those of saline-injected AR2 mice (Saline) (n=3), $*p < 0.05$ (Mann -Whitney U-test). All error bars represent the s.e.m.

B.C. Immunoblot analysis of brain (B) and spinal cord extracts (C) demonstrated that the expression of ADAR2 protein did not significantly differ between AAV and Saline groups.

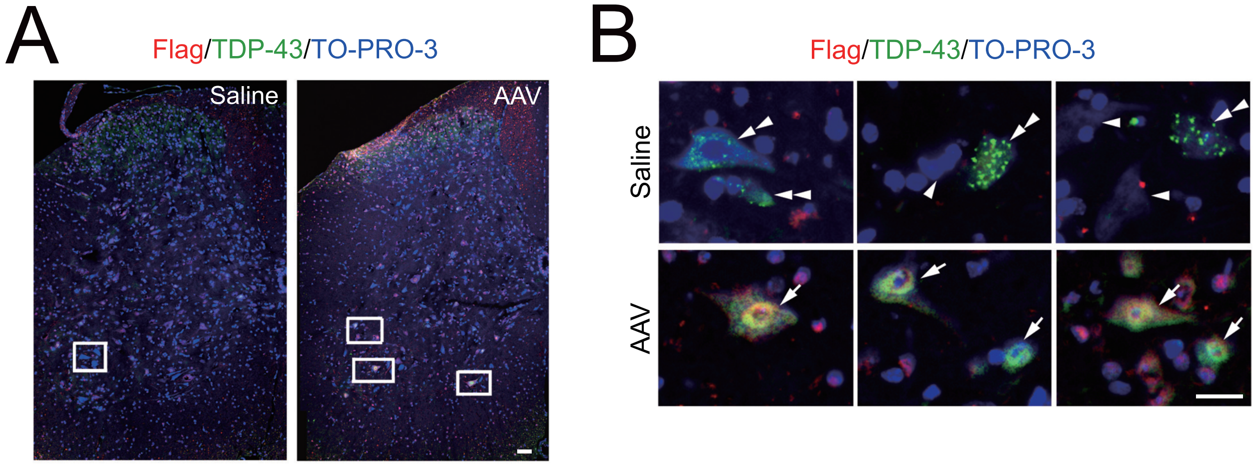

Supporting Information Figure S9. Rescue of TDP-43 in motor neurons.
A. Immunohistochemistry for TDP-43 (green) and Flag (red) in the right half of the spinal cord of AR2 mice intravenously injected with AAV9-Flag-hADAR2. The boxed areas represent the area for high power magnification image of Figure 5A. Scale bar, 50 µm.
B. There were AHCs devoid of TDP-43 immunoractivity (arrowheads) and those exhibiting numerous TDP-43-positive cytoplasmic aggregates without nuclear TDP-43 immunoreactivity (double arrowheads) in the saline-treated AR2 mice (Saline). Flag-positive AHCs exhibited intense nuclear and faint cytoplasmic TDP-43 immunoreactivity (arrows) in AAV9-Flag-ADAR2-injected AR2 mice (AAV). Scale bar is 20 µm.

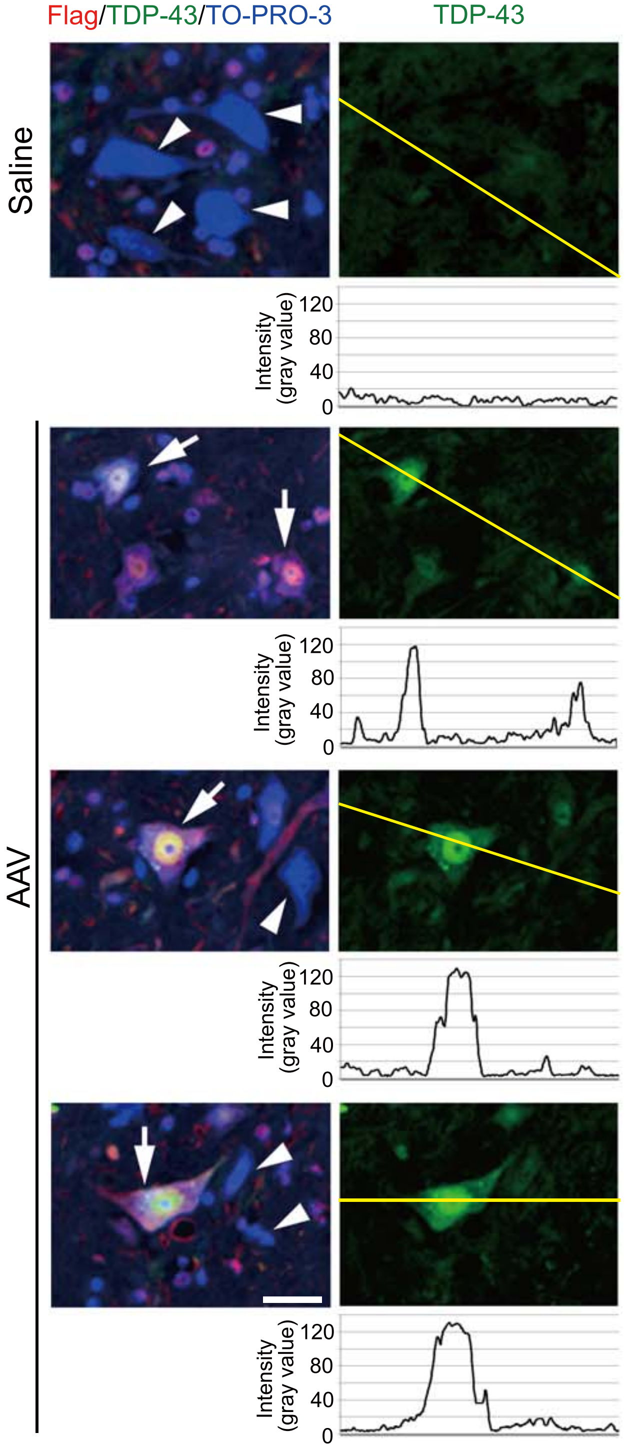

Supporting Information Figure S10. The signal intensity of TDP-43-positive and -negative AHCs.

Immunohistochemistry for TDP-43 (green) and Flag (red) in the spinal cord. AHCs were recognized as large (≥ 20 μm diameter) and TO-PRO-3-positive (blue) cells in the AH. The panels represent the enlarged image of Figure 5A. The profile images showed fluorescence intensity of TDP-43 along the yellow line. In Figure 5C, we designated AHCs as TDP-43-positive when the signal intensity was more than 3-fold higher (> 60) than the background (average ± s.e.m., 8.80 ± 0.12; max 20).

Table S1. Primer sequences.

		oligonucleotide sequence
human GluA2 mRNA	hG2F2	5'-TTCCTGGTCAGCAGATTTAGCC-3'
	hG2R2	5'-TTCCCTTTGGACTTCCGCAC-3'
	hG2F4	5'-TCTGGTTTTCCTTGGGTGCC-3'
	hG2R9	5'-AGATCCTCAGCACTTTCG-3'
human GluA2 pre-mRNA	hG2F2	5'-TTCCTGGTCAGCAGATTTAGCC-3'
	hG2IR1	5'-GCAACATTCAAAGAACATTGTTC-3'
	hG2F4	5'-TCTGGTTTTCCTTGGGTGCC-3'
	hG2IR2	5'-CCGAAGCTAAGAGGATGTCCTTC-3'
mouse GluA2 mRNA	hG2F2	5'-TTCCTGGTCAGCAGATTTAGCC-3'
	mG2R2	5'-TTCCCTTTGGATTTCCTGAC-3'
	hG2F4	5'-TCTGGTTTTCCTTGGGTGCC-3'
	hG2R9	5'-AGATCCTCAGCACTTTCG-3'
mouse GluA2 mRNA (spinal cord)	mGluA2-F(2119)	5'-GAGTGGCACACTGAGGAATTTGAAG-3'
	mGluA2-R(2362)	5'-GACACCATCCTCTCTACAGTCAGGA-3'
mouse GluA2 pre-mRNA	hG2F2	5'-TTCCTGGTCAGCAGATTTAGCC-3'
	mG2IR1	5'-TCAACATTCAAGGAACATTGTTC-3'
	hG2F4	5'-TCTGGTTTTCCTTGGGTGCC-3'
	mG2IR2	5'-GCCGACGGTAAAAATTTGCCTTC-3'
mouse CYFIP2 mRNA	mCYFIP2 F6	5'-GAGCTGCTGGCAGACATTGTCAAC-3'
	mCYFIP2 R6	5'-CCGGTACTCTTCATCCGACTTCTGG-3'
mouse CYFIP2 mRNA (brain)	mCYFIP2 F	5'-GCACATGTTGCTCAAGGTGATGGGC-3'
	mCYFIP2 R	5'-TGTTGCTGTAGCGAGCCAGCTCAGA-3'
Flag-human-ADAR2	FLAG-F1	5'-CAAGGATGACGACGATAAGATC-3'
	hADAR2-R1	5'-CTTGATCTCATTCAGCTGCATC-3'
GFP	GFP-F1	5'-ACGTAAACGGCCACAAGTTCAGCGT-3'
	GFP-R1	5'-CGGACTTGAAGAAGTCGTGCTGCTT-3'
	GFP-F3	5'-TAAACGGCCACAAGTTCAGCGTGTC-3'
	GFP-R3	5'-AGAAGTCGTGCTGCTTCATGTGGTC-3'

Table S2. TaqMan probes used for quantitative polymerase chain reaction.

		oligonucleotide sequence	Universal Probe Library
mouse GluA2 mRNA	mLC106F	5'-AAACAGAAATTGCTTATGGAACATT-3'	probe: #106 (Roche cat. no. 04692250001)
	mLC106R	5'-CAAACACTGCAATTTTAGATCTCC-3'	
mouse ADAR2	mLC42F	5'-ATCCCGCCTGTGTAAGCA-3'	probe: #42 (Roche cat. no.04688015001)
	mLC42R	5'-GACTCGTGGTATGTGGTAGGC-3'	
mouse β-actin	mLC64F	5'-CTAAGGCCAACCGTGAAAAG-3'	probe: #64 (Roche cat. no.04688635001)
	mLC64R	5'-ACCAGAGGCATACAGGGACA-3'	
human GluA2 mRNA	hLC63F	5'-ATGCGATATTTCGCCAAGA-3'	probe: #63 (Roche cat. no. 04688627001)
	hLC63R	5'-CAGTCAGGAAGGCAGCTAAGTT-3'	
human ADAR2	hLC42F	5'-GTGTAAGCACGCGTTGTACTG-3'	probe: #42 (Roche cat. no.04688015001)
	hLC42R	5'-CGTAGTAAGTGGGAGGGAACC-3'	
human and mouse ADAR2	hmLC87F	5'-GCGGATCTCCAACATAGAGG-3'	probe: #87 (Roche cat. no.04689127001)
	hmLC87R	5'-CCAGTTGACACTGAAGTTGGG-3'	
